# Reading and writing of mRNA m^6^A modification orchestrate maternal-to-zygotic transition in mice

**DOI:** 10.1186/s13059-023-02918-9

**Published:** 2023-04-06

**Authors:** Wencheng Zhu, Yufeng Ding, Juan Meng, Lei Gu, Wenjun Liu, Li Li, Hongyu Chen, Yining Wang, Ziyi Li, Chen Li, Yidi Sun, Zhen Liu

**Affiliations:** 1 Institute of Neuroscience, CAS Center for Excellence in Brain Science and Intelligence Technology, CAS Key Laboratory of Primate Neurobiology, State Key Laboratory of Neuroscience, Chinese Academy of Sciences, Shanghai, China; 2 Shanghai Center for Brain Science and Brain-Inspired Intelligence Technology, Shanghai, China; 3 University of Chinese Academy of Sciences, Beijing, China; 4 Center for Single-Cell Omics, School of Public Health, Shanghai Jiao Tong University School of Medicine, Shanghai, China; 5 Shanghai Applied Protein Technology Co., Ltd., Shanghai, China

**Keywords:** m^6^A landscape, Multi-omics, Maternal-to-zygotic transition, Ythdc1, Ythdf2

## Abstract

N^6^-methyladenosine (m^6^A) modification has been shown to regulate RNA metabolism. Here, we investigate m^6^A dynamics during maternal-to-zygotic transition (MZT) in mice through multi-omic analysis. Our results show that m^6^A can be maternally inherited or de novo gained after fertilization. Interestingly, m^6^A modification on maternal mRNAs not only correlates with mRNA degradation, but also maintains the stability of a small group of mRNAs thereby promoting their translation after fertilization. We identify Ythdc1 and Ythdf2 as key m^6^A readers for mouse preimplantation development. Our study reveals a key role of m^6^A mediated RNA metabolism during MZT in mammals.

**Supplementary Information:**

The online version contains supplementary material available at 10.1186/s13059-023-02918-9.

## Background

Maternal-to-zygotic transition (MZT) is a fundamental and conserved process, during which the maternal environment of the oocyte transitions to the zygotic genome driven expression program. This process reprograms the terminally differentiated oocyte and sperm to a state of totipotency [1–4]. MZT is initiated by maternal mRNAs and proteins that are present during the period of zygotic genome quiescence after fertilization. This is followed by a gradual switch to the activation of the zygotic genome and is accompanied by the clearance of maternal RNAs and proteins [3–6]. A key question for embryonic development is how the MZT is regulated. Recent studies have illustrated a series of epigenetic reprogramming involved in MZT, including global DNA demethylation and methylation, chromatin remodeling, genome reorganization, and downstream transcriptional changes [2, 7, 8]. However, despite the significance of maternally deposited yet rapidly cleared mRNAs and newly synthesized zygotic RNA products in driving early embryogenesis [1, 4], the various mechanisms of RNA control, such as posttranscriptional regulation and specific RNA epigenetic regulation, remain poorly understood.

N^6^-methyladenosine (m^6^A) is the most prevalent internal modification present in the messenger RNA (mRNA) across higher eukaryotes [9, 10]. It affects the stability and translation of modified transcripts, providing a mechanism for coordinated regulation of groups of transcripts mediated by writer, eraser, and reader proteins during cell fate maintenance and transitions [11–13]. In mammals, m^6^A has been shown to regulate embryonic stem cell differentiation through modifications on key mRNAs or transposable element RNAs, evidenced by the early embryonic lethality of m^6^A writers or readers knockout (KO) mice, including Ythdc1 and Ythdf2, or Mettl3 [14–18]. In zebrafish, previous report showed that abundant and reversible mRNA m^6^A can promote the decay of a subset of maternal mRNAs dependent on the reader Ythdf2 [19], suggesting a possible role of posttranscriptional RNA modification in the global regulation of maternal mRNA metabolism. However, due to the limited amounts of oocytes and preimplantation embryos of mice, the potential regulation role of m^6^A modification on RNA metabolism during MZT remains largely unknown. Here, we revealed the m^6^A dynamics during mouse MZT by a combined analysis of multi-omics data generated or adopted from low-input profiling methods, and reveals an important role of m^6^A in early embryo development.

## Results and discussion

**Fig. 1:**
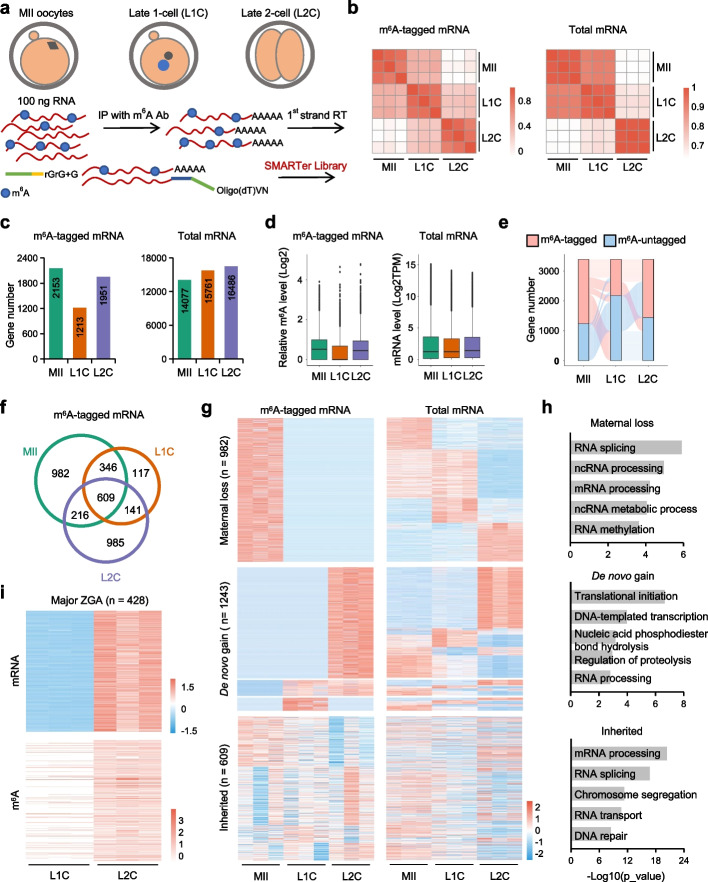
Comprehensive m^6^A landscape during mouse MZT. **a** Schematic diagram of sample collection and SLIM-seq procedure. **b** Heatmap showing the pairwise correlations of m^6^A and mRNA expression profiles. **c** The numbers of m^6^A-tagged mRNAs and total mRNAs in different stages. **d** Boxplot plots displaying the log2 normalized m^6^A levels and mRNA expression levels in different stages. **e** The Sankey diagram showing the dynamics of m^6^A tagged mRNAs. Red represents the gene defined as m^6^A-tagged mRNAs cross different stages of MZT. **f** Venn diagram showing overlapping of m^6^A tagged genes from different stages. **g** Heatmap showing normalized m^6^A levels and the corresponding mRNA expression levels in different stages. **h** Gene ontology (GO) enrichment analysis of three types of the m^6^A tagged genes. For maternal loss, 453 genes with decrease at both m^6^A and mRNA levels are used for analysis. For de novo gain, 628 genes with increase at both m^6^A and mRNA levels are used for analysis. **i** Heatmap showing normalized m^6^A levels and the corresponding mRNA expression levels in ZGA

To reveal the dynamic m^6^A landscape of mRNA during maternal-to-zygotic transition (MZT) in mice, we used a recently developed SLIM-seq strategy (Fig. 1a) as it is suitable for low-input samples [20], such as mammalian oocytes and embryos. To evaluate the sensitivity and selectivity of the SLIM method, an m^6^A RNA immunoprecipitation (MeRIP) was performed using an RNA mixture containing equal amounts of an m^6^A-modified control RNA (green fluorescent protein, GFP) and an unmodified control RNA (mCherry). The GFP RNA was transcribed in vitro in the presence of 20% m^6^ATP and 80% ATP as reported before [21]. Quality control analysis by quantitative real-time PCR (qRT-PCR) showed that 50 ng input RNA libraries exhibited relatively high signal-to-noise (SN) ratios of spike-in RNAs GFP (m^6^A^+^)/mCherry (m^6^A^−^) (Additional file 1: Fig. S1a). MII oocytes as well as late 1-cell (L1C) and late 2-cell (L2C) stage embryos were collected and used for SLIM-seq analysis, while the corresponding RNA-seq analysis was performed on RNA samples from the same batches but without immunoprecipitation (IP). The experiment was performed in triplicates for each stage, and all replicates demonstrated high reproducibility in both the SLIM-seq and RNA-seq datasets (Fig. 1b). MII oocytes and L1C embryos showed high similarity in the mRNA profiles but were distinguished by m^6^A profiles. We identified a total of 3396 m^6^A-tagged mRNAs and 973 m^6^A-tagged ncRNAs from SLIM-seq (Fig. 1b and Additional file 1: Fig. S1b). The numbers of m^6^A-tagged mRNAs significantly decreased after fertilization while increased during zygotic genome activation (ZGA), indicating a highly dynamic nature of m^6^A in early events of new life (Fig. 1c). Consistently, the m^6^A levels altered in the same way as the number of m^6^A-tagged mRNAs in different stages (Fig. 1d). Comparative analysis revealed three major patterns of m^6^A dynamics during early embryonic development, including maternal lost, consistent inherited and de novo gain (Fig. 1e and Additional file 2: Table S1). Very recently, a similar profile of m^6^A dynamics during MZT was reported using a method called ultralow-input (ULI) m^6^A RNA immunoprecipitation (MeRIP) followed by sequencing (ULI-MeRIP–seq) [20]. We compared our m^6^A profile with this work, and found that we both identified a large number of the same genes with m^6^A modification (Additional file 1: Fig. S1c-e). We noticed that Wu et al*.* showed that the number of m^6^A^+^ transcripts increased after fertilization [22]. This might be due to the differences in the used oocyte/embryo number, mouse genetic background or ways of fertilization. Together, these results describe a highly dynamic m^6^A landscape of mRNAs during mouse MZT.

Oocyte maturation and fertilization are accompanied by a small and a major wave of RNA degradation, respectively. In mice, approximately 20% of maternal RNAs undergo active degradation during oocytes maturation, and RNA m^6^A reader Ythdf2 was shown to be essential for maternal RNA decay and oocyte competence [14]. Whether m^6^A is involved in the major wave of RNA degradation after fertilization is largely unknown. Through comparative analysis, we found 982 m^6^A-tagged genes specifically enriched in MII oocytes and lost after fertilization (Fig. 1f). Besides, nearly half of these genes showed decreased expression at mRNA level, indicating a pro-decay role of m^6^A on these mRNAs (Fig. 1g, upper panel). Gene ontology (GO) enrichment analysis showed that these genes are enriched for ncRNA metabolic process and RNA splicing (Fig. 1h, upper panel). From L1C to L2C, 1243 genes were newly m^6^A-tagged and were thus considered as de novo generated after fertilization, especially at L2C stage, which reminds us a key event of early embryonic development, i.e., the major ZGA. As illustrated in Fig. 1g (middle panel), about half of the m^6^A-tagged genes showed a similar increased level in mRNA expression, suggesting that about half of the de novo modification of m^6^A is accompanied with zygotic transcription. In line with our expectation, we detected 428 m^6^A-tagged genes during major ZGA and about 400 genes showed evidently de novo m^6^A modification (Fig. 1i). The other 3031 ZGA genes are not detected m^6^A mark in our profiling (Additional file 1: Fig. S1f). Besides, the 628 out of 1243 genes that were de novo generated m^6^A and transcriptionally increased are significantly enriched in important events that are required for early development, such as translational initiation, regulation of proteolysis and RNA processing (Fig. 1h, middle panel).

**Fig. 2:**
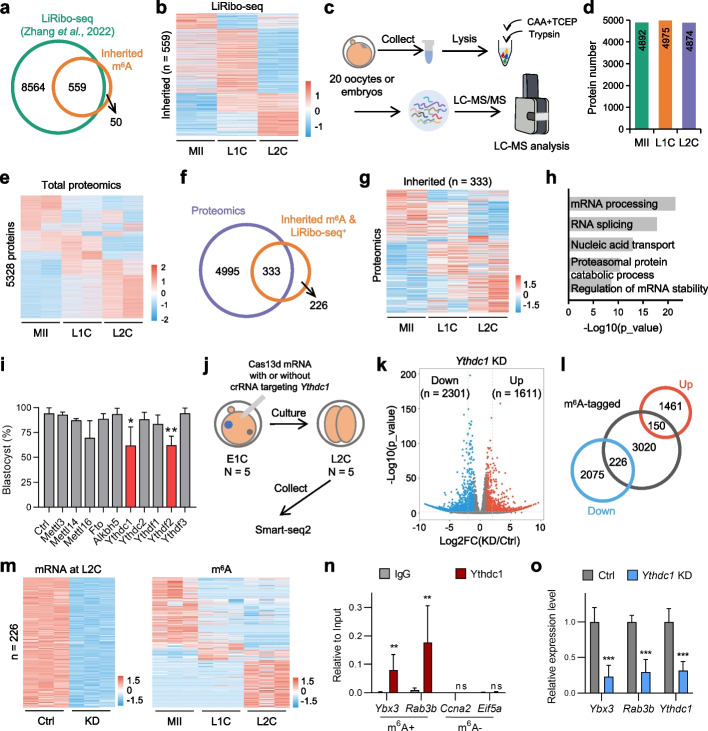
A group of maternally inherited m^6^A maintains mRNA stability and relates to protein translation. **a** Venn diagram showing overlap between LiRibo-seq detected genes and inherited m^6^A-tagged genes. **b** Heatmap showing the relative gene expression levels by LiRibo-seq that overlapped with inherited m^6^A tagged genes. **c** Workflow of low-input proteomic analysis. 20 oocytes or embryos were pooled and transferred into low adsorption tube, then lysed, digested, and analyzed by ultra-sensitive LC–MS/MS. Both hybrid spectral library and final DIA (data-independent acquisition) data were generated by Spectronaut™. **d** The number of protein groups detected in different stages. **e** Heatmap showing the relative protein expression levels in different stages. **f** Venn diagram showing the overlap between LiRibo-seq detected genes and proteins identified by proteomic data in oocytes and preimplantation embryos. **g** Heatmap showing the relative expression levels of 333 proteins detected by MS. **h** GO enrichment analysis of genes shown in (G). **i** Blastocyst ratios in the control and the indicated gene knockdown groups. Ctrl represents Cas13d mRNA only. Data represent the mean ± standard deviation (SD) of three biologically independent experiments (two-tailed Student’s t-test). **P < 0.01, *P < 0.05. **j** Schematic diagram of sample collection and processing. E1C, early 1-cell. **k** Volcano plot displaying the DEGs between Ctrl and *Ythdc1* KD groups at L2C stage. The DEGs are identified with *P* value < 0.05 and absolute log2 fold-change > 1. **l** Venn diagram showing the overlap between DEGs and the m^6^A-tagged genes. **m** Heatmaps showing the relative expression levels of downregulated genes from m^6^A-marked genes after *Ythdc1* KD (left) and their corresponding m^6^A levels (right). **n** RIP-qPCR showing Ythdc1 associated m^6^A-tagged mRNAs. Obtained signals were normalized to the Input. IgG and m^6^A-untagged genes served as negative controls. Data represent the mean ± SD of three biologically independent experiments (two-tailed Student’s t-test). **P < 0.01, ns denotes not significant. **o** qRT-PCR results of indicated mRNAs that immunoprecipitated with Ythdc1 after *Ythdc1* KD. *Ythdc1* was measured to verify the KD efficiency. Obtained signals were normalized to *Actb*, and were relative to Ctrl. Data represent the mean ± standard deviation of three biologically independent experiments (two-tailed Student’s t-test). ***P < 0.001

Interestingly, we also observed 609 m^6^A-tagged genes that are consistently inherited from MII oocytes to L2C embryos. However, similar to m^6^A, no obvious pattern at mRNA level was observed (Fig. 1g, lower panel). In addition to facilitating RNA decay, m^6^A modification also involves in maintaining RNA stability [12, 23]. As these modified mRNAs are inherited, we speculated that these mRNAs are maintained for translation. Therefore, we explored whether these genes were actively translated using a recently published LiRibo-seq dataset at the similar developmental stages (Additional file 1: Fig. S2a-c) [24]. Comparative analysis revealed that that out of the 609 m^6^A-tagged genes, 559 were also detected in LiRibo-seq data (Fig. 2a and Additional file 2: Table S1). In addition, we analyzed other mRNAs, including total mRNAs, total m^6^A-untagged mRNAs (m^6^A^−^) and total m^6^A-tagged mRNAs (m^6^A^+^). For the genes that can be detected by LiRibo-seq, we referred them as “translation active” while the undetected ones are “translation silent”. As illustrated in Additional file 1: Fig. S2d, the inherited genes showed the highest translation active ratio (559/609, ~ 91.8%) when comparing to total (9010/17387, ~ 51.8%), m^6^A^−^ (6470/13991, ~ 46.2%) or m^6^A^+^ (2540/3396, 74.8%) genes. However, if we randomly select 609 genes, the proportion of actively translated genes is much lower during MZT compared to the m^6^A-tagged genes (Additional file 1: Fig. S2e). Besides, the majority of the 559 m^6^A-tagged genes also showed elevated translation levels at 1-cell and 2-cell stages (Fig. 2b), indicating that these maternally inherited m^6^A-marked mRNAs undergo translation at these stages. Furthermore, we observed that the proportion of actively translated genes among the 559 m^6^A-tagged genes is comparable to that of genes detected and activated at the 1-cell stage during MZT, but significantly higher than that of m^6^A-negative genes or non-inherited m^6^A-positive genes (Additional file 1: Fig. S2f). The 609 inherited genes showed similar levels of m^6^A modification as the "maternal loss" or "de novo gain" genes (Additional file 1: Fig. S2g). Moreover, they were more likely to be inherited than the genes without the m^6^A mark (Additional file 1: Fig. S2h).

To further verify our hypothesis that the inherited m^6^A facilitates translation, we utilized our recently developed ultrasensitive proteome technology to examine the proteomic dynamics during MZT (Fig. 2c) [25]. Using only 20 oocytes or embryos per replicate for the proteomic analysis, this method enabled the reproducible detection of 5353 proteins coded by 5328 genes (Fig. 2d-e and Additional file 1: Fig. S3a-c), which were at least detected in both replicates of any stage. Notably, this level of protein detection is comparable to the latest embryonic proteomic resource generated from 8000 mouse embryos. (Additional file 1: Fig. S3d-e) [26]. Over 80% of detected proteins showed active translation by comparison with the detected genes of LiRibo-seq data (Additional file 1: Fig. S3f). Out of the 559 genes detected by both SLIM-seq and LiRibo-seq, a total of 333 genes were also identified by our low-input proteomics approach (Fig. 2g and Additional file 2: Table S1). Moreover, a heatmap of the 333 genes revealed that the majority of them were either maintained or increased in expression levels during MZT. GO analysis showed that these genes are enriched for mRNA and proteasome regulation (Fig. 2h). Therefore, our data suggested that the maternally inherited m^6^A-tagged mRNAs are maintained for translation.

Although we have observed the orchestrated m^6^A dynamics during MZT, the extent to which m^6^A-mediated RNA metabolism contributes to early embryo development, as opposed to oocyte maturation, remains largely unknown. Mettl3, Mettl14 and Mettl16 are m^6^A writers, while Fto and Alkbh5 are m^6^A erasers. These epigenetic codes on RNAs are recognized by readers including Ythdc1, Ythdc2, Ythdf1, Ythdf2 and Ythdf3. To investigate the dynamics of translation and transcription of these genes, we first analyzed the published LiRibo-seq and RNA-seq data [27] during mouse preimplantation development (Additional file 1: Fig. S4a-b). Our analysis revealed that several genes, including *Mettl14*, *Fto*, and *Ythdc2*, were both transcriptionally and translationally silent. In contrast, *Mettl3*, *Ythdc1*, and *Ythdf3* exhibited high translational activity during MII and 1C stages. Furthermore, *Mettl16* and *Ythdf2* were actively transcribed and involved in translation, particularly during the 2C to 4C stages of early embryo development. We also explored these genes in our proteomic data. As shown in Additional file 1: Fig. S4c, Mettl3, Fto, Alkbh5, Ythdc1, Ythdc2, Ythdf2 and Ythdf3 were detected in our proteomic data, highlighting the sensitivity of this approach. These findings indicate that different m^6^A-related genes may play distinct roles during various stages of early embryonic development. Next, we applied the recently developed CRISPR-Cas13d to knockdown (KD) m^6^A related regulators in mouse zygotes [28]. Specifically, the pronuclear (PN) 1 stage zygotes were used for Cas13d/crRNA injection. As the expression levels of these genes are diverse, we selected specific time points ranging from 6 h post injection to E2.0 to assess the efficacy of gene knockdown (Additional file 1: Fig. S4d). After microinjection, the embryos were cultured for the examining of blastocyst formation. We found that the targeted mRNAs were efficiently knocked down and the blastocyst rate is significant reduced in the embryos with *Ythdc1* or *Ythdf2* KD (Fig. 2i and Additional file 1: Fig. S4e). Recently, Chen et al*.* demonstrated that CRISPR/Cas9-mediated *Ythdc1* KO at zygote stage did not affect blastocyst formation but reduced hatching [29]. We used CRISPR/Cas9 to knock out *Ythdc1* and obtained the similar results (Additional file 1: Fig. S4f-h). As illustrated in Additional file 1: Fig. S4a-b, the transcription level of Ythdc1 is relatively low but its translation is active from MII to 4C stages. Therefore, the data above indicated that the maternally-inherited portion of *Ythdc1* mRNA plays a role in preimplantation development, however these mRNAs cannot be depleted by Cas9. Ivanova et al*.* used conditional KO (cKO) of *Ythdf2* in oocytes to establish an essential role of Ythdf2 for oocyte maturation in mouse [14]. They further found that *Ythdf2* cKO resulted in developmental failure at the 2C stage. We observed an evident increase of Ythdf2 at both transcription and translation levels at major ZGA as revealed by RNA-seq and LiRibo-seq data (Additional file 1: Fig. S4a-b). How does Ythdf2 expression at major ZGA affect preimplantation development is unknown. Thus, our results here discriminate the roles of Ythdf2 in oocyte maturation and early development.

*Ythdc1* is actively translated and transcribed before L2C stage, and an increase of its protein level was also detected (Additional file 1: Fig. S4c). Therefore, we speculated that Ythdc1 played a role during MZT. To investigate the effect of Ythdc1 in transcriptome level, we microinjected *Ythdc1* targeted Cas13d/crRNA into early zygotes and collected the resulted L2C stage embryos for transcriptome analysis using Smart-seq2 (Fig. 2j and Additional file 1: Fig. S5). We identified 3912 differentially expressed genes (DEGs) between control (Ctrl, Cas13d mRNA only) and *Ythdc1* KD embryos (Fig. 2k). Among these down-regulated genes, about 10% (226/2301) genes are m^6^A-tagged (Fig. 2l). It is presumable that Ythdc1 may affect the stability of these m^6^A-marked mRNAs. As shown in Fig. 2m, loss of Ythdc1 leads to the down-regulation of 226 genes with m^6^A mark, suggesting a role of maintaining RNA stability. To test whether Ythdc1 involved in mRNA stability regulation, we performed RNA immunoprecipitation (RIP)-qPCR assay based on several selected candidates from the above-mentioned 226 genes. As shown in Fig. 2n and Additional file 1: Fig. S6a, m^6^A-tagged mRNAs, including *Ybx3*, *Rab3b*, *Commd3*, *Camk1d* and *Tcea18*, are associated with Ythdc1. Additionally, qRT-PCR further validated that knockdown of *Ythdc1* reduced the mRNA levels of these genes (Fig. 2o and Additional file 1: Fig. S6b). We used Actinomycin D (ActD) to inhibit the transcription of these genes followed by knockdown of *Ythdc1*(Additional file 1: Fig. S6c), so that we can test whether Ythdc1 involves in regulating mRNA stability instead of transcription or splicing. As shown in Fig. S6d, *Ythdc1* was evidently knocked down at 4 h post microinjection, and ActD was effective for transcription inhibition as *Ythdc1* should be upregulated at L1C stage (PN5) as shown above. All five of the tested genes exhibited increased RNA decay following *Ythdc1* knockdown. (Additional file 1: Fig. S6e). Collectively, we preliminarily concluded that Ythdc1 associated with m^6^A-tagged genes and involved in their stability regulation. A recent study demonstrated that YTHDC1 can modulate autophagy *via* regulating the stability of *SQSTM1* mRNA in diabetic keratinocytes [30]. In sum, we evaluated the roles of ten key m^6^A regulators in mouse preimplantation development and uncovered how Ythdc1 influence the fate of m^6^A-marked mRNAs.

## Conclusions

Oocyte maturation and fertilization are accompanied by a small and a major wave of RNA degradation, respectively. In mice, approximately 20% of maternal RNAs undergo active degradation during oocytes maturation, and it has been shown that the RNA m^6^A reader Ythdf2 is essential for maternal RNA decay and oocyte competence [14]. However, whether m^6^A is involved in the major wave of RNA degradation after fertilization is largely unknown. Through comparative analysis of RNA-seq and SLIM-seq data, we observed a correlation between maternal RNA decay and m^6^A modification. Most maternally inherited RNAs are dedicated to decay rapidly, while some mRNAs are transcribed into proteins for ZGA which is considered as the first transcription event in life. By integrating SLIM-seq, LiRibo-seq, low-input proteomics and RIP-qRT-PCR data, we demonstrated that a small group of maternal mRNAs with m^6^A mark are maintained throughout the MZT, and most of these genes are actively translated, indicating a role of m^6^A in safeguarding RNA stability for early embryo development.

The concept of m^6^A mediated maternal RNA degradation has been verified in zebrafish [19]. Recently, it has been observed that de novo m^6^A modification occurs during mammalian ZGA [22]. We have independently confirmed this using the SLIM-seq method [20], and found that a large number of mRNAs undergo de novo m^6^A modification after fertilization, with the majority of modifications taking place at the major ZGA. However, these findings raise several questions that need to be addressed in future studies. For instance, how does de novo m^6^A modification affects the fates of marked ZGA mRNAs? Additionally, why are there many non-ZGA mRNAs that are marked with m^6^A? Are these mRNAs newly transcribed or maternally inherited from the zygotes? Finally, how does m^6^A regulate the fate of these mRNAs?

Several studies have demonstrated the crucial role of m^6^A-mediated RNA metabolism in oocyte development and maturation [14, 31, 32], but its involvement in early development remains elusive. To address this, we used CRISPR-Cas13d to systemically evaluate the roles of ten m^6^A regulators in mouse preimplantation development and identified Ythdc1 and Ythdf2 as key regulators. Surprisingly, knockdown of m^6^A writers did not significantly affect early development, even though we detected numerous de novo generated m^6^A signals. This might be due to the sufficient storage of writer proteins in MII oocytes. For example, Mettl3 has been demonstrated to be essential for oocyte maturation and its depletion at germinal vesicle (GV) stage greatly hampered early embryonic development [32, 33]. However, as the transcription and translation of *Mettl3* and *Mettl14* are evidently decrease after fertilization, it is also possible that there are other unknown writers involved in de novo modification. Although we found that the depletion of *Fto* did not affect preimplantation development, Wei et al*.* recently revealed that Fto mediates m^6^A demethylation of long-interspersed element-1 (LINE1) RNA, thereby shaping chromatin state and gene expression during oocyte and post-implantation development in mice [34]. In brief, the diverse functions of m^6^A regulators in different developmental stages of mouse oocytes and embryos, including but not limited to Ythdc1, Ytddf2, Mettl3 and Fto, as we and others observed, highlight a delicate and sophisticated regulation of RNA metabolism orchestrated by m^6^A.

## Methods

### Reagents and antibodies

Pregnant mare serum gonadotropin (PMSG, 110914564) and human chorionic gonadotropin (hCG, 110911282) were purchased from San-sheng Biotechnology. Hyauronidase (H4272), Cytochalasin B (CB, C6762), HTF medium (MR-070-D), M2 medium (M7167), bovine albumin (BSA, A7030), KSOM medium (MR-101-D) and NP-40 (I3021) were obtained from Sigma-Aldrich. TRIzol (15596–026) was purchased from Invitrogen. Protein A magnetic beads (88845) and mMESSAGE mMACHINE™ T7 Transcription Kit (AM1344) were obtained from Thermo Fisher Scientific. AMPure XP beads (A63881) were purchased from Beckman. RNasin, TruePrep® DNA Library Prep Kit V2 for illumina Kit (TD501) and ChamQ SYBR Color qPCR Master Mix (Without ROX) (Q321) were purchased from Vazyme. RNA Clean Beads (N243-01B), T7 High Yield RNA Transcription Kit (E131) and NLS-Cas9 Nuclease (E365-01A) were purchased from Novoprotein. KAPA HiFi HotStart ReadyMix (KK2601) was obtained from Roche. Primary antibodies were used against: m^6^A modification (ab15320, abcam), Ythdc1 (14392–1-AP, Proteintech), β-Actin (A5441, Sigma-Aldrich). Actinomycin D (ActD, HY-17559) was obtained from MedChemExpress.

### Collection of mouse oocytes and embryos

Mouse MII oocytes were derived from wild-type B6D2F1 (C57BL/6 J x DBA/2N) strain female mice [35]. For superovulation, 8-week-old female mice were injected intraperitoneally with 10 IU of PMSG, followed by the injection of 10 IU hCG 48 h later. MII oocytes were collected from oviducts of the superovulated female mice 14 to 16 h after hCG injection, and cumulus cells were removed from oocytes by briefly incubating in M2 medium with hyaluronidase.

For late 1 cell and late 2 cell stage embryo collection, superovulated B6D2F1 female mice were mated with adult B6D2F1 males, and zygotes were collected from female oviducts at 14 to 16 h post-hCG injection. Zygotes with two PN were transferred from M2 medium to KSOM and cultured at 37 °C under 5% CO_2_. Late 1 cell and late 2 cell stage embryos were collected after hCG injection for 27 to 29 h and 48-50 h, respectively.

For ActD treatment, zygotes were collected at PN3 stage and transferred into KSOM containing 5 μg/mL ActD. The subsequent microinjection and culture were conducted with 5 μg/mL ActD.

All cultured embryos were tested negative for mycoplasma.

### In vitro transcription of Cas13d mRNA, sgRNA, or crRNA

T7 promoter was added to N terminus of Cas13d coding region by PCR amplification from Cas13d expression vector, using the indicated primers (Additional file 3: Table S2). T7-Cas13d PCR production was purified and used as the template for in vitro transcription (IVT) using mMESSAGE mMACHINE™ T7 Transcription Kit. T7 promoter was added to sgRNA/crRNA DNA templates by PCR amplification as previous reported [28, 36], with primers listed in Additional file 3: Table S2. The T7-sgRNA/crRNA PCR products were purified and used as the DNA templates for IVT using T7 High Yield RNA Transcription Kit. Both the mRNA and sgRNA/crRNA were purified using RNA Clean Beads according to the manufacturer’s protocols.

### Preparation of injection mixtures

All injection mixtures were prepared in a final volume of 10 μL according to the following protocol. Using RNase-free water, reagents, and consumables. For Cas13d-mediated gene KD, Cas13d mRNA (final concentration 300 ng/μL) was mixed with three different crRNAs (final concentration 300 ng/μL per crRNA) for one target gene. For Cas9-mediated gene KO, Cas9 protein (final concentration 100 ng/μL) was mixed with three different sgRNAs (final concentration 100 ng/μL per sgRNA) for one target gene.

### Mouse embryo injection and culture

All injection mixtures were prepared in a final volume of 10 μL according to the following protocol. Cas13d/crRNA or Cas9/sgRNA mixture was injected to the cytoplasm of zygote before well recognized pronuclei at the volume of 1–3 pL. Microinjection were performed in a droplet of M2 medium containing 5 μg/mL CB using a Piezo-driven micromanipulator (Prime Tech). Then, the injected embryos were cultured in KSOM medium with amino acids at 37 °C under 5% CO_2_ in air until E4.5.

### Quantitative real-time PCR (qRT-PCR)

qRT-PCR experiments were performed using the Roche480 II Real-Time PCR System (Roche) and ChamQ SYBR Color qPCR Master Mix (Without ROX) according the manufacturer’s instructions. The signal obtained from *Actb* mRNA was used as a loading control for normalization. The primers used are shown in Additional file 3: Table S2.

### RNA-seq library preparation and sequencing

The RNA-seq library was prepared using our customized protocol based on Smart-seq2 [37]. Total RNA were reverse transcribed with VN-anchored oligo-dT primer and TSO. The resulting full-length cDNA is preamplified with the ISPCR primers by KAPA HiFi HotStart ReadyMix and purified using AMPure XP beads to abolish primer dimers. Pre-amplified cDNA was fragmented by Tn5 enzyme, followed by library generation using TruePrep® DNA Library Prep Kit V2 for illumina Kit and were pair-end sequenced on Illumina HiSeq X Ten sequencing platform with paired-ended 150-bp reads.

### SLIM-seq

SLIM-seq was performed as previously reported [20]. The procedure was as follows: Total RNA was isolated using TRIzol reagent. Anti-m^6^A antibody (0.5 μg per sample) was pre-bound to Protein A magnetic beads in IP buffer (10 mM Tris–HCl (pH 7.4), 100 mM NaCl,0.1% NP40, 0.4 U/μL RNasin) with head-to-tail mixing at 4 °C for 2 h. Total RNA (100 ng per sample) was added and incubated at 4 °C for another 2 h. Beads were washed twice with 200 μL IP buffer, twice with 200 μL low-salt buffer (10 mM Tris–HCl (pH 7.4), 50 mM NaCl, 0.1% NP-40, 0.4 U/μL RNasin), twice with 200 μL high-salt buffer (10 mM Tris–HCl (pH 7.4), 500 mM NaCl, 0.1% NP40, 0.4 U/μL RNasin) and once with 200 μL 1 × IP buffer. Captured RNA was eluted by heating beads for 2 min at 94 °C in 10 μL DEPC H_2_O. The library was prepared using customized protocol based on Smart-seq2. The sequence of TSO primer for template switching is AAGCAGTGGTATCAACGCAGAGTACATrGrG + G. After reverse transcription, the cDNAs were pre-amplified by 13 cycles of PCR using the ISPCR primers (AAGCAGTGGTATCAACGCAGAGT). After purification by AMPure XP beads, 50 ng pre-amplified cDNA was fragmented by Tn5 enzyme, followed by library generation using TruePrep® DNA Library Prep Kit V2 for illumina Kit and the libraries were sequenced on Illumina Hiseq X Ten platform in 150 bp paired-end manner.

### Spike-in controls for MeRIP-qRT-PCR

m^6^A RNA immunoprecipitation (MeRIP)-qRT-PCR was performed using an RNA mixture containing equal amounts of an m^6^A-modified control RNA (green fluorescent protein, GFP) and an unmodified control RNA (mCherry). The GFP RNA control was transcribed in vitro in the presence of 20% m^6^ATP and 80% ATP, as established by a previous report [21]. For each MeRIP, 0.05 ng of GFP and mCherry RNA were spiked into 50 ng of total RNA extracted from MII zygotes, similar to a previous study [22]. Subsequent procedures are same as the above-mentioned SLIM-seq and qRT-PCR protocols. The calculation of SN ratio was performed as described before [21].

### SLIM-seq data analysis

SLIM-seq data analysis was performed following a previous study [20]. Specifically, R package tximport [38] was used to estimate the read counts at gene level from the alignment files obtained by kallisto [39]. Then, we normalized the read counts for each gene by total reads in each sample and gene length to eliminate the effect of different library sizes, and transformed counts matrix to TPM matrix. The TPM values were used for the following analysis. To calculate the relative m^6^A level for each gene, we applied the DESeq2 to compare the gene counts between IP samples and input samples on a transcriptome-wide scale. Based on the adjusted fold-change of each gene from the output of DESeq2, and the adjusted fold-change in each embryonic stage was used as the relative m^6^A level. We identified high-confidence m^6^A-tagged genes by setting up the thresholds as follows, read count > 1 in input samples, log2 fold-change for IP versus Input > 0, and *P* value < 0.05; If the *P* value equals ‘‘NA’’ in DESeq2 output, we identified high-confidence m6A-tagged genes as log2 fold-change > 2. We kept the m^6^A genes identified in at least two of the three replicates in at least one of the three stages for the downstream analysis.

### RNA-seq and LiRibo-seq data analysis

For RNA-seq data from *Ythdc1* knockdown and control experiments, the raw sequencing was processed with Trimmomatic (v0.39) [40] to remove low quality sequences and adaptors (LEADING:3 TRAILING:3 MINLEN:50). Qualified reads were mapped to the reference genome (GRCm38.99) by Hisat2 (v2.2.1) and the read counts for each transcript were quantified by featureCounts (v2.0.1) [41]. We applied DESeq2 to identify differentially expressed genes between groups, and the significant genes were identified with *P* value < 0.05 and log2 fold-change > 1. For data presentation, we also calculated transcripts per million (TPM) and log2 normalized for each gene. We downloaded the LiRibo-seq and RNA-seq data from GSE169632 [42], and kept the genes detected in two replicates of at least one of three stages.

### RNA Immunoprecipitation (RIP)

Native RIP was performed following a previous protocol with some modifications [43]. In brief, Protein A beads (10 μL) were washed with IP buffer (10 mM Tris–HCl (pH 7.4), 150 mM NaCl, 0.1% NP40) and incubated with anti-Ythdc1 antibody (2 μg) and 5 μL short primer (100 μM) with head-to-tail mixing at 4℃ for 2 h. L2C embryos (n = 150, triplicate) were collected and washed three times by PBS, then split into two aliquots, one was moved into IP buffer, 10% sample were moved into input-lysis buffer. Embryos lysed on ice for 20 min with vertexing. Samples in IP buffer containing RNase inhibitor and protease inhibitors were added into antibody-beads complex and incubated at 4℃ for another 3 h. Beads were washed sequentially with 200 μL IP buffer for three times and wash buffer (10 mM Tris–HCl (pH7.4), 50 mM NaCl, 0.1% NP40) for three times. The captured RNAs were eluted in 10 μL DEPC H_2_O by heat elution (95 °C for 2 min). The library was prepared using customized protocol based on Smart-seq2 as described above.

### Western blotting

One hundred fifty embryos at L2C were washed with PBS and collected. Samples were lysed following a published protocol [44]. Western blotting was performed as described before [45].

### Proteomic data analysis

The proteomic data was obtained by liquid chromatography with tandem mass spectrometry (LC–MS-MS), and normalized with upper quantile. We kept the genes detected in two replicates in at least one of three stages for the downstream analyses.

### Minor ZGA and major ZGA analysis

We identified minor ZGA genes by setting up the fold-change of TPM for L1C versus MII samples > 5. Likewise, major ZGA genes were defined by the fold-change of TPM for L2C versus L1C samples > 5.

### Gene enrichment analysis

Differentially expressed genes were performed functional enrichment analyses including GO terms and KEGG pathways using R package clusterProfiler.

### Proteomic preparation

Twenty oocytes or embryos were collected for one replicate and prepared following our ultrasensitive proteomic workflow [25]. In brief, cells were lysed in lysis buffer at 60 °C for 2 h, then CAA and TCEP were added at final concentrations of 40 mM and 10 mM, respectively. After incubation for 5 min at 95 °C, reduced and alkylated proteins were incubated with pre-washed magnetic beads (Fisher Scientific, Germany) in 50% EtOH for 15 min, then the protein-binding beads were washed thrice with 80% EtOH and trypsinized in 100 mM ABC buffer at 37 °C for 4 h. The supernatant containing digested peptides was acidized and transferred to a C18 homemade StageTip for desalting. Clean peptides were lyophilized and resuspended in 0.1% (v/v) formate/ddH_2_O for LC–MS/MS analysis.

### LC–MS/MS analysis

The LC–MS/MS analysis was performed on a trapped ion mobility spectrometry coupled to time-of-flight mass spectrometer (timsTOF MS, Bruker) combined with a high performance applied chromatographic system nanoElute® (Bruker). Peptides were loaded on to an in-house packed column (75 μm × 250 mm; 1.9 μm ReproSil-Pur C18 beads, Dr. Maisch GmbH, Ammerbuch) which was heated to 60 °C, and separated with a 60-min gradient of 2% to 80% mobile phase B at a flow rate of 300 nL/min. The mobile phases A and B were 0.1% (v/v) formate/ddH_2_O and 0.1% (v/v) formate/acetonitrile, respectively. The mass spectrometer was performed in a data-independent acquisition (DIA) parallel accumulation-serial fragmentation (PASEF) mode [46]. Fragment analysis was subdivided into 64 × 26 Th precursor isolation windows from m/z 400 to 1200 with 1 Th isolation width overlap. The collision energy was ramped linearly as a function of the mobility from 59 eV at 1/K_0_ = 1.6 Vs cm^−2^ to 20 eV at 1/K_0_ = 0.6 Vs cm^−2^.

### Database searching

Mass spectrometry raw files were processed in Spectronaut™ (version 16) with default settings. A hybrid spectral library of mouse oocyte and preimplantation embryo proteome which contained 140511 precursors, 96036 peptides and 6632 protein groups was used as the searching spectral library.

### Statistical analysis

All the statistical analysis was performed using R programming language. We used the “cor” function in R to calculate the Pearson’s correlation between each pair of samples. The "t.test" was used to perform two-sample Student’s t tests, the "fisher.test" was used to performed Fisher's exact test for testing the null of independence of rows and columns in a contingency table with fixed marginals, and the "chisq.test" was used to performed chi-squared contingency table tests and goodness-of-fit tests. The *P* value was adjusted with FDR or Benjamini–Hochberg correction for multiple comparisons. The ggplot2 package (v3.3.1) was used to generate most of the plots.

## Supplementary Information

**Additional file 1.** Supplementary figures and figure legends (Fig. S1-S6).

**Additional file 2:** **Table S1.** The processed SLIM-seq, RNA-seq and proteomics data.

**Additional file 3:** **Table S2.** Defined gene list, sgRNA sequence, crRNA sequence and primer sequence used for qRT-PCR.

**Additional file 4.** Uncropped western blot images. Related to Fig. S4h in Additional file 1.

**Additional file 5.** Review history.

## Data Availability

Sequencing data for SLIM-seq (IP and Input) and RNA-seq have been deposited in the Gene Expression Omnibus under the accession number GSE228201 [47]. The mass spectrometry proteomics data have been deposited to the ProteomeXchange Consortium via the PRIDE [48] partner repository with the dataset identifier PXD041023 [49]. Of note, each stage contains three replicates, and after quality control, MII_1, MII_2, 1cell_1, 1cell_2, late2_2 and late2_3 were usd for analysis. Previously published LiRibo-seq and RNA-seq data [24] used in this study can be found in the Gene Expression Omnibus under the accession number GSE169632 [42]. No custom scripts and software were used other than those mentioned in the Methods section.
