# Supplementary material for: Reading and writing of mRNA m6A modification orchestrate maternal-to-zygotic transition in mice
Source: Genome Biol. 2023 Apr 6;24:67. doi: 10.1186/s13059-023-02918-9 (PMC10080794; doi:10.1186/s13059-023-02918-9)
Supplement: Supplementary file 1 — Additional file 1. Supplementary figures and figure legends (Fig. S1-S6). [file 13059_2023_2918_MOESM1_ESM.pdf]

Reading and writing of mRNA m^{6}A modification orchestrate maternal-to-zygotic transition in mice

Authors

Wencheng Zhu, Yufeng Ding, Juan Meng, Lei Gu, Wenjun Liu, Li Li, Hongyu Chen, Yining Wang, Ziyi Li, Chen Li, Yidi Sun, Zhen Liu

Supplementary figures and figure legends (S1-S6).

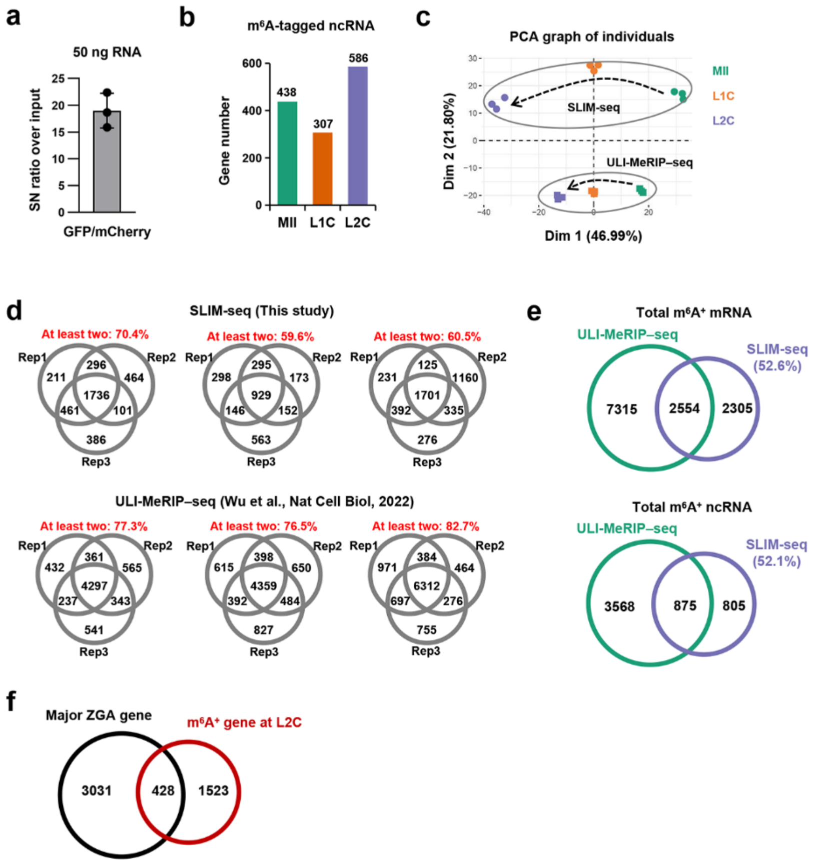

Fig. S1.

(a) High enrichment of m6A in IP samples over input with 50 ng total RNA tested by qPCR of GFP (m^6A^+) versus mCherry (m^6A^-). Data are presented as mean ± SD of three biologically independent experiments. SN, signal-to-noise.

(b) The numbers of m^6A-tagged ncRNAs in different stages.

(c) Principal component analysis (PCA) of two independent m^6A profiling studies.

(d) Venn diagram showing the overlap of identified m^6A-tagged genes among three replicates from two independent studies using two different m^6A profiling methods. Both mRNA and ncRNA are included.

(e) Venn diagram showing the overlap of identified m^6A-tagged genes between two independent studies.

(f) Venn diagram showing the overlap between major ZGA genes and m^6A^+ genes at L2C.

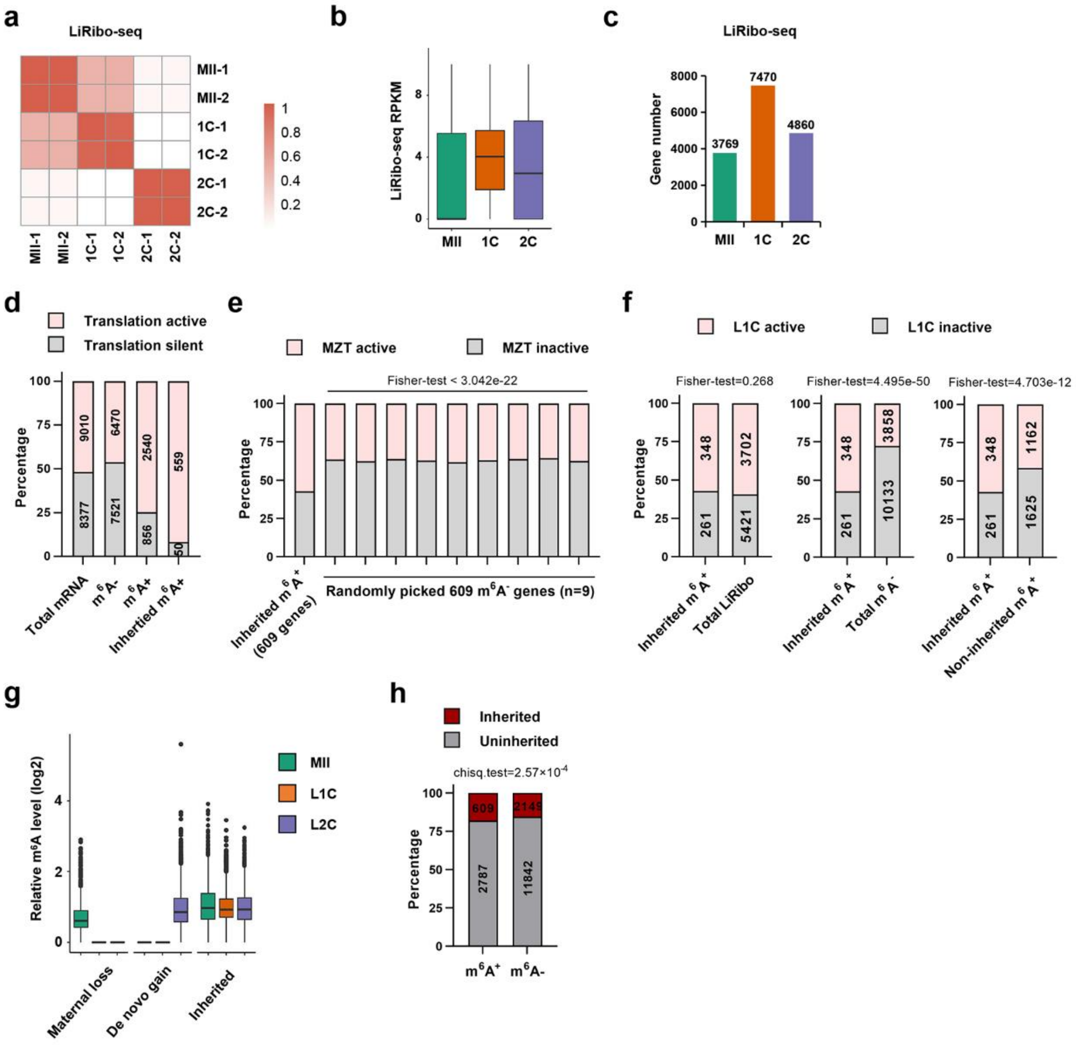

Fig. S2.

(A) Heatmap showing the pairwise correlations of gene expression profiles of LiRibo-seq data.

(B) Boxplot plot showing the distribution of gene expression levels (RPKM) quantified by LiRibo-seq in different stages.

(C) The numbers of detected genes in different stages.

(D) Proportion of transcription active genes from different groups of mRNAs.

(E) Proportion of MZT active mRNAs in 609 inherited m^6A^+ genes or randomly picked m^6A^- genes. Two-tailed Fisher's exact test was used to calculate the *P*-value.

(F) Proportion of L1C active mRNAs in 609 inherited m^6A^+ genes or other indicated groups of genes. Two-tailed Fisher's exact test was used to calculate the *P*-value.

(G) Boxplot showing the log2 normalized m^6A levels (TPM) of genes among the 3 groups at different developmental stages.

(H) Comparison of the ratio of inherited and uninherited genes between m^6A^+ and m^6A^- mRNA genes. The *P*-value was calculated with Chi square test.

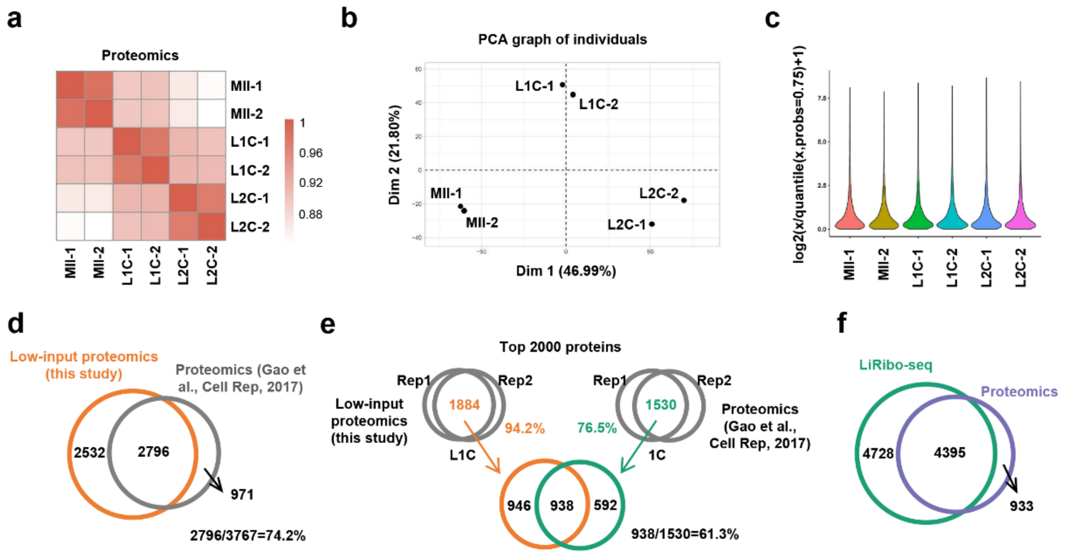

Fig. S3.

(a) Heatmap showing the pairwise correlations of protein expression profiles quantified by proteomic data.

(b) Principal component analysis (PCA) of proteomic data.

(c) Violin plot displaying the log2 normalized protein levels per sample in different stages.

(d) Venn diagram showing the overlap of identified proteins between two studies.

(e) Venn diagram showing the overlap of identified proteins among two replicates from two different studies at L1C (left) or 1C (right) stages. The reproducibly identified proteins were further compared between the two studies.

(f) Venn diagram showing the overlap between LiRibo-seq detected genes and proteins identified by low-input MS in oocytes and preimplantation embryos.

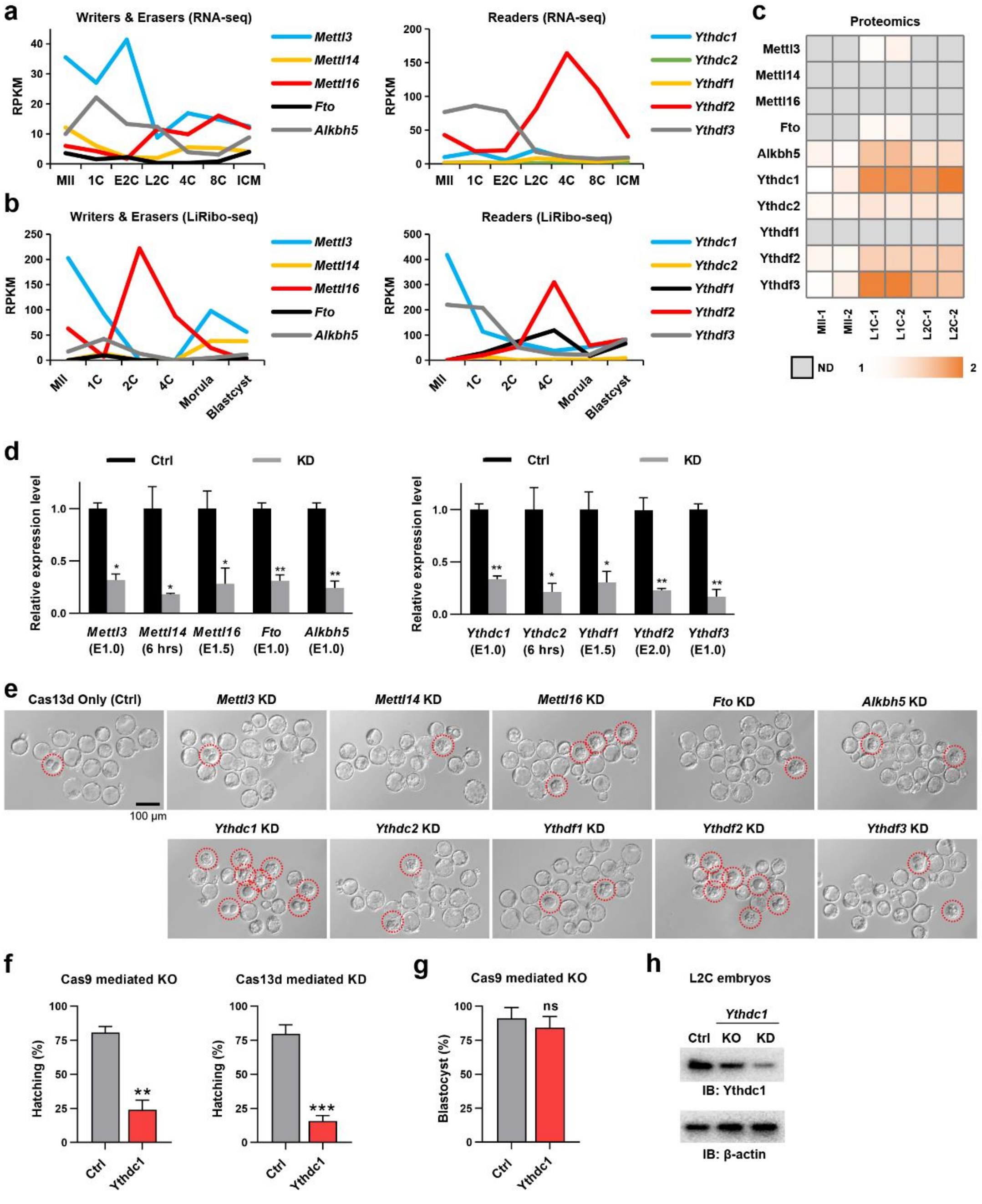

Fig. S4.

(a) Expression patterns of ten m^6A regulators during early embryonic development.

(b) Ribosome binding patterns of ten m^6A regulators during early embryonic development.

(c) Protein levels of ten m^6A regulators in different stages.

(d) qRT-PCR results of indicated genes after microinjection of targeting crRNAs and Cas13d mRNA. Obtained signals were normalized to *Actb*, and were relative to values of Ctrl. Data represent the mean ± standard deviation of three biologically independent experiments (two-tailed Student's t-test). ***P* < 0.01, **P* < 0.05.

(e) Representative photos of mouse embryos after CRISPR-Cas13d RNA editing targeting different genes at E4.0.

(f) Hatching ratios in the Ctrl and *Ythdc1* KO or KD groups. Ctrl represents Cas9 protein only (left) or *Cas13d* mRNA only (right). Data represent the mean ± standard deviation (SD) of three biologically independent experiments (two-tailed Student's t-test). $**P < 0.01$, $***P < 0.001$.
(g) Blastocyst ratios in the control and *Ythdc1* KO groups. Data represent the mean ± standard deviation (SD) of three biologically independent experiments (two-tailed Student's t-test). ns denotes not significant.
(h) Cas9-mediated KO or Cas13d-mediated KD on *Ythdc1* was measured by western blotting. β-actin served as loading control.

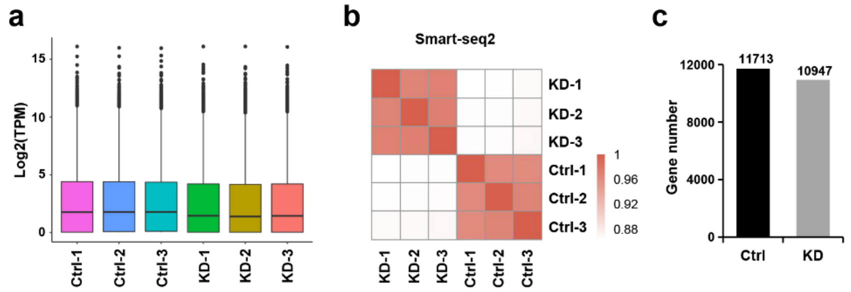

Fig. S5.

(a) Boxplot plot displaying the RNA expression levels in different samples. Ctrl, Cas13d mRNA only; KD, *Ythdc1* knockdown.

(b) Heatmap showing the pairwise correlation of gene expression profiles in Ctrl and KD groups.

(c) The numbers of detected genes in Ctrl and KD groups.

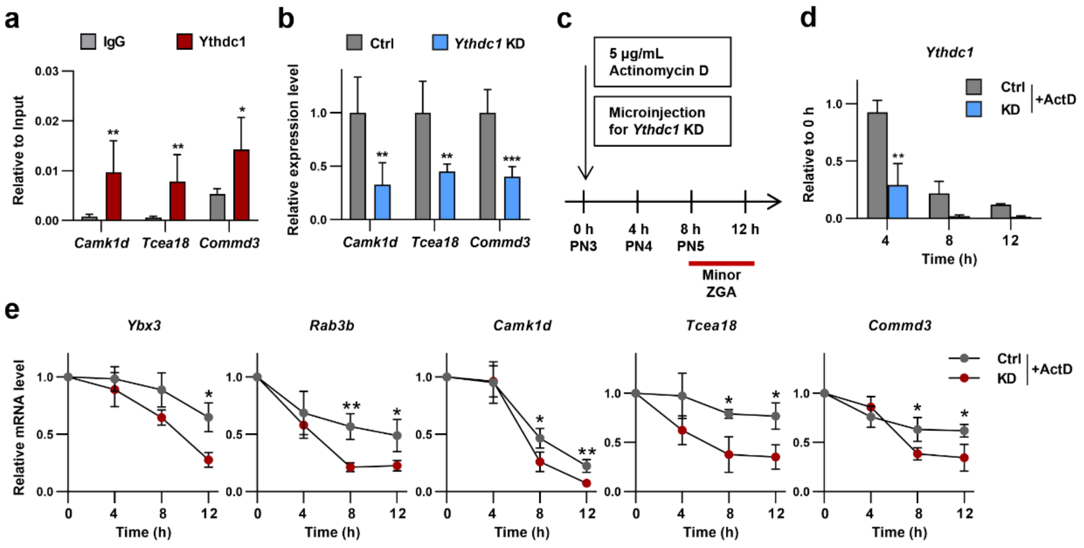

Fig. S6.

(a) RIP-qPCR showing Ythdc1 associated m^{6}A-tagged mRNAs. Obtained signals were normalized to the Input. IgG served as negative controls. Data represent the mean ± SD of three biologically independent experiments (two-tailed Student's t-test). *$P < 0.05$, **$P < 0.01$.

(b) qRT-PCR results of indicated mRNAs that immunoprecipitated with Ythdc1 after *Ythdc1* KD. Obtained signals were normalized to *Actb*, and were relative to Ctrl (Cas13d mRNA only). Data represent the mean ± standard deviation of three biologically independent experiments (two-tailed Student's t-test). **$P < 0.01$, ***$P < 0.001$.

(c) The workflow of Actinomycin D (ActD) treatment and microinjection in early zygotes. Samples were collected at indicated time points and used for qRT-PCR assay.

(d) qRT-PCR results of *Ythdc1* mRNA levels after Cas13d-mediated *Ythdc1* KD. Obtained signals were normalized to *Actb*, and were relative to values at 0 h. Data represent the mean ± standard deviation of three biologically independent experiments (two-tailed Student's t-test). **$P < 0.01$.

(e) The mRNA levels of indicated genes were measured by qRT-PCR after ActD treatment and *Ythdc1* KD. Obtained signals were normalized to *Actb*, and were relative to values at 0 h. Data represent the mean ± standard deviation of three biologically independent experiments (two-tailed Student's t-test). *$P < 0.05$, **$P < 0.01$.
