# Supplementary figures and images for: Reading and writing of mRNA m6A modification orchestrate maternal-to-zygotic transition in mice
Source: Genome Biol. 2023 Apr 6;24:67. doi: 10.1186/s13059-023-02918-9 (PMC10080794; doi:10.1186/s13059-023-02918-9)

Fig. S4h

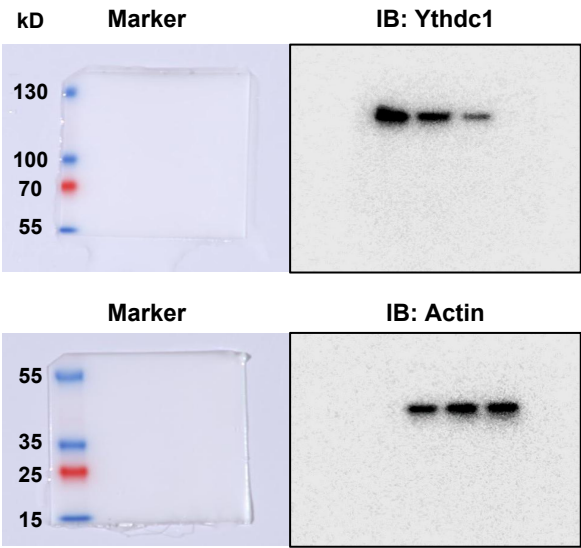

Supplement: Supplementary file 4 — Additional file 4. Uncropped western blot images. Related to Fig. S4h in Additional file 1. [file 13059_2023_2918_MOESM4_ESM.pdf]
