# Supplementary material for: Reading and writing of mRNA m6A modification orchestrate maternal-to-zygotic transition in mice
Source: Genome Biol. 2023 Apr 6;24:67. doi: 10.1186/s13059-023-02918-9 (PMC10080794; doi:10.1186/s13059-023-02918-9)
Supplement: Supplementary file 5 — Additional file 5. Review history. [file 13059_2023_2918_MOESM5_ESM.docx]

Review History

**First round of review**

**Reviewer 1**

**Were you able to assess all statistics in the manuscript, including the appropriateness of statistical tests used?**

Yes, and I have assessed the statistics in my report.

**Comments to author:**

In this manuscript, Zhu, et al. utilized m6A-seq, LiRibo-seq and low-input proteomic analysis to reveal the dynamic profiles of m6A and proteomics during mouse MZT. Author raised the m6A promotes the stabilization and translation of m6A-inherited mRNAs through integrated multi-omics analysis. Moreover, author used Cas13d to KD the key m6A regulators and found Ythdc1 and Ythdf2 have impact on early embryo development. Overall, the study is challenging and interesting with some new findings. However, some conclusions need to be strengthened with more evidence.

Major comments

1. Through integrated multi-omics analysis, author found the maternally inherited m6A tagged mRNAs are maintained for translation. This conclusion cannot to be done with current evidence. There are about 600 inherited m6A tagged mRNAs based on SLIM-seq in Fig2A and Fig2B. And 95 genes displayed positive correlation between translation and protein level in Fig2K with only half of them have increased translation trend during MZT. This result seems to be a random result, which means when I randomly pick 600 m6A unmodified genes, there may also be more genes with increased translation level during MZT? It is an interesting point that m6A promotes the translation of ZGA transcripts or maternally inherited-m6A transcripts, but what else data analysis can prove the conclusion?

2. In Fig2P, author found there are only 44 de novo gained m6A genes are downregulated in Ythdc1 KD embryos. It is not strong evidence to explain Ythdc1 have a role of maintaining RNA stability for m6A. On the other hand, how many downregulated genes after Ythdc1 KD are unmodified by m6A? There are no evidence showed Ythdc1 prefer to maintain RNA stability for m6A-marked genes.

Specific comments

1. Since Wu et al.1 have recently published m6A modified genes during mouse MZT using ULI-MeRIP-seq. How many m6A modified genes are overlapped between these two individual studies?

2. In fig 2F, author showed the LiRibo-seq dynamics of 559 inherited m6A genes. It is interesting that most of them are activated at L1C when the minor wave ZGA happened. How many genes are activated at L1C in the whole genes detected by LiRibo-seq ? is it a higher ratio in inherited m6A genes? May be do a fisher test to show the significance.

3. The phenotype of YTHDC1 KD using Cas13d is not quite identical to the YTHDC1 KO in zygote in previously published paper2 (Chuan Chen et al., Protein & Cell, 2021) which have normal blastocyst rate but hatching problems. What's the differences? Did you test the protein level of YTHDC1 after KD assay?

4. METTL3 was recently found to ensure the timely decay the 2C-specific m6A transcripts according to Wu et al .1 Several studies about the m6A function in embryos and embryonic stem cells are recently published1-3. Citation in the manuscript need to be updated.

Reference:

1 Wu, Y. et al. N(6)-methyladenosine regulates maternal RNA maintenance in oocytes and timely RNA decay during mouse maternal-to-zygotic transition. Nat Cell Biol 24, 917-927, doi:10.1038/s41556-022-00915-x (2022).

2 Chen, C. et al. Nuclear m(6)A reader YTHDC1 regulates the scaffold function of LINE1 RNA in mouse ESCs and early embryos. Protein Cell 12, 455-474, doi:10.1007/s13238-021-00837-8 (2021).

3 Wei, J. et al. FTO mediates LINE1 m(6)A demethylation and chromatin regulation in mESCs and mouse development. Science 376, 968-973, doi:10.1126/science.abe9582 (2022).

**Reviewer 2**

**Were you able to assess all statistics in the manuscript, including the appropriateness of statistical tests used?**

No, I do not feel adequately qualified to assess the statistics.

**Comments to author:**

The manuscript "Reading and writing of mRNA m6A modification orchestrate mouse 2 maternal-to-zygotic transition" is a good effort to map changes in epitranscriptomic mark, m6A on maternal and zygotic RNA to understand the role of most prevalent RNA modification in zygotic genome activation. Particularly, authors have used the low input multi-omics strategies to reveal role of m6A during early phase of development. The low input multi-omics approaches can solve problem of the material required to study early development in mammals. Maternal-to-zygotic (MZT) transition involves enormous changes in the transcriptomic landscape, thus, it is plausible that the epitranscriptomic marks will change too and can play pivotal role in MZT. So far, no one has studied this systematically except one recent paper (Wu NCB 2022).

Here, the authors report the following conclusions:

1) Maternal m6A is inherited.

2) Newly transcribed mRNA in zygotes are m6A methylated.

3) Ythdf2 mediates m6A's function in mRNA decay and clearance.

4) Besides mediating mRNA degradation, m6A mediates mRNA stabilisation and promotes mRNA translation.

5) Ythdc1 has a role in stabilizing m6A methylated mRNAs.

The conclusions of this manuscript are either purely based on correlation (4,5) or not notable (1-3). I will expand on my criticism of these conclusions.

Maternal m6A is inherited:

There is poor evidence showing that m6A is demethylated after entering the cytoplasm since any potential demethylase is secluded to the nucleus. Therefore, if maternal mRNAs are inherited, it is not surprising that maternal m6A is also inherited.

Newly transcribed mRNA in zygotes are m6A methylated:

Since Mettl3 is present in zygotes, it is not surprising that newly transcribed mRNAs gain m6A. This is akin to any newly transcribed mRNA being spliced or gaining a mRNA cap.

Ythdf2 mediates m6A's function in mRNA decay and clearance:

This has already been demonstrated in mice (Ivanova 2017 Mol. Cell)

Besides mediating mRNA degradation, m6A mediates mRNA stabilisation and promotes mRNA translation:

The evidence that the authors provide here are simply only correlative. The authors did not attempt any reverse genetics of m6A readers or Mettl3 to probe the effect of m6A on mRNA translation. Instead, they only show that for a subset of m6A mRNA, there is correlation between presence of m6A and active translation. Furthermore, the proteomics result are similar to other studies (Gao et al., Cell reports 2017) and here, they found no new hits.

Ythdc1 has a role in stabilizing m6A methylated mRNAs:

Past published results have only demonstrated that IGF2BP proteins mediate stabilisation of m6A mRNA. While the authors did knock down Ythdc1, they focused only on the subset of 44 down-regulated genes out of 376 genes that are both differentially expressed and possess m6A, in order to reach the conclusion that Ythdc1 functions in stabilizing these m6A mRNAs. At the very least, they will need some kind of crosslinking-IP experiment to show that Ythdc1 binds to these m6A mRNAs to stabilize them, which was not performed.

With the following major concerns, I believe that in present form the manuscript is not suitable for Genome Biology.

Other comments:

1- The sample size to infer most results is low: 50 oocytes or embryos per replicates are not sufficient to make mechanistic conclusions.

2- In addition, it will be great if authors can compare the results of LiRibo-seq with a recently published study where they profiled mRNA translation during mouse MZT (Zhang et al,. Science Adv. 2022) and low input m6A transcripts result with another recent study Wu et al,. 2022 (NCB).

3- The legends should be consistent and the presentation of results should be coherent, at least in the same figure. Inconsistent legends are bothersome for the readers and difficult to compare. For example in Figure 1B for m6A-Seq, the sequence of the results are arranged differently than RNA Seq.¬

4- The quality of the figures should be improved and presentation should be clear. Moreover, the resolution of figures, particularly Figure 2 is quite low.

**Authors Response**

**Point-by-point responses to the reviewers’ comments:**

Reviewer #1:

In this manuscript, Zhu, et al. utilized m6A-seq, LiRibo-seq and low-input proteomic analysis to reveal the dynamic profiles of m6A and proteomics during mouse MZT. Author raised the m6A promotes the stabilization and translation of m6A-inherited mRNAs through integrated multi-omics analysis. Moreover, author used Cas13d to KD the key m6A regulators and found Ythdc1 and Ythdf2 have impact on early embryo development. Overall, the study is challenging and interesting with some new findings. However, some conclusions need to be strengthened with more evidence.

Major comments 1. Through integrated multi-omics analysis, author found the maternally inherited m6A tagged mRNAs are maintained for translation. This conclusion cannot to be done with current evidence. There are about 600 inherited m6A tagged mRNAs based on SLIM-seq in Fig2A and Fig2B. And 95 genes displayed positive correlation between translation and protein level in Fig2K with only half of them have increased translation trend during MZT. This result seems to be a random result, which means when I randomly pick 600 m6A unmodified genes, there may also be more genes with increased translation level during MZT? It is an interesting point that m6A promotes the translation of ZGA transcripts or maternally inherited-m6A transcripts, but what else data analysis can prove the conclusion?

*Response: We thank the reviewer for the constructive suggestions. To response the concerns from the reviewer, we performed more analysis and provided additional data to strengthen our conclusion.*

*1) We further analyzed the translation degrees of total mRNA, m6A+ mRNA, m6A- mRNA and inherited m6A+ mRNA (for convivence, named as “inherited”). We referred genes that can be detected by LiRibo-seq as translation active, and the undetected ones as translation silent. As illustrated in new Fig S2A, inherited genes showed the highest translation active ratio (559/609, ~91.8%) in comparison with total (9010/17387, ~51.8%), m6A- (6470/13991, ~46.2%) or m6A+ (2540/3396, 74.8%) genes.*

*2) In response to the Reviewer’s suggestion, we randomly picked 609 m6A unmodified genes for nine times (sampling analysis), and found that less than 40% of these randomly selected genes were MZT active, significantly lower than that observed in the inherited m6A tagged genes (55 %; new Fig S2B).*

*3) In the previous manuscript, we only showed 95 genes from the 559 inherited m6A+ & LiRibo+ genes as they displayed a positive correlation between translation and protein level. However, we realized that the relative m6A levels may not coincide with the increase of proteins as protein translation lags behind mRNA, so the m6A heatmap was removed. Among the 559 genes that are both translation active and inherited, we detected 333 genes in our proteomics data (new Fig 2F), and most of them showed consistent or increased protein levels (new Fig 2G). Of note, the DIA database used for protein searching was updated recently, and we obtained more proteins from our low-input proteomic analysis (from a total number of 4317 to 5328).*

2. In Fig2P, author found there are only 44 de novo gained m6A genes are downregulated in Ythdc1 KD embryos. It is not strong evidence to explain Ythdc1 have a role of maintaining RNA stability for m6A. On the other hand, how many downregulated genes after Ythdc1 KD are unmodified by m6A? There are no evidence showed Ythdc1 prefer to maintain RNA stability for m6A-marked genes.

*Response: We thank the reviewer for the kind suggestions. Previously, we mainly focused on the impact of Ythdc1 KD on a small group of mRNAs, i.e., de novo gained m6A. We acknowledged that old Fig 2P might be a little misleading, thus we have rearranged these data. As the Reviewer can see, there are totally 226 genes with m6A mark (~10%) among the total down-regulated genes (now presented as new Fig 2L and Fig 2M). There are more down-regulated genes without m6A mark which might be an indirect regulation. It is important to note that this assumption is in agreement with a recent report (Wu et al., Nat Cell Biol, 2022) in which a key component of m6A methyltransferase complex, Kiaa1429, mediates m6A to stabilize Z-decay (decay after ZGA) mRNAs in oocytes, while among the 1869 down-regulated genes after conditional knock out of Kiaa1429, there are 282 genes marked with m6A (~15%), and most of them are indirectly regulated.*

*To confirm that Ythdc1 involves in maintaining RNA stability, we performed RNA immunoprecipitation (RIP)-qPCR assay based several selected candidates from the above-mentioned 226 genes. As shown in new Fig 2N and Fig S4D, m6A-marked mRNAs, including Ybx3, Rab3b, Commd3, Camk1d and Tcea18, are associated with Ythdc1. Additionally, qPCR further validated that knockdown of Ythdc1 reduced the mRNA levels of these genes (new Fig 2O and Fig S4E).*

*Collectively, we concluded that Ythdc1 is associated with m6A marked genes and involved in their stability regulation. Very recently, a study also demonstrated that YTHDC1 can modulate autophagy via regulating the stability of SQSTM1 mRNA in diabetic keratinocytes (Liang et al., Autophagy, 2022).*

Specific comments 1. Since Wu et al.1 have recently published m6A modified genes during mouse MZT using ULI-MeRIP-seq. How many m6A modified genes are overlapped between these two individual studies?

*Response: In response to the reviewer’s suggestion, we have compared the m6A modified genes in the two studies. Even though the two studies used different mA6-seq methods (SLIM-seq vs ULI-MeRIP-seq), sample numbers (100~250 oocytes or embryos vs 540~750 oocytes or embryos), mice background (B6D2F1 vs C57BL/6) and ways of fertilization (in vivo vs in vitro), we both identified a large number of m6A modified genes, and more than 50% of m6A modified genes identified in this study were overlapped with the previous study (new Fig S1B and S1C). The PCA analysis further verified a similar pattern of the m6A dynamics during MTZ (new Fig S1D), even though a batch effect is observed (Dim 2).*

*In short, the two studies generally confirmed each other independently, but the transcriptomic data obtained from different experimental conditions and methods showed evident batch effects that rendered their in-depth comparison.*

2. In fig 2F, author showed the LiRibo-seq dynamics of 559 inherited m6A genes. It is interesting that most of them are activated at L1C when the minor wave ZGA happened. How many genes are activated at L1C in the whole genes detected by LiRibo-seq? is it a higher ratio in inherited m6A genes? May be do a fisher test to show the significance.

*Response: Thanks for the kind suggestion. We performed more analysis as the reviewer suggested. We found, as shown in new Fig S2C, the translation ratio of inherited genes is similar to L1C activated genes. This is reasonable that a large number of genes start to translation after fertilization (as illustrated in Fig S1G). A key conclusion drew from the LiRibo-seq paper (Zhang et al., Sci Adv, 2022) is that the authors identified n significant increase and transition of translation from MII oocytes to zygotes and demonstrated that active translation of maternal mRNAs is essential for maternal-to-zygotic transition (MZT). However, the inherited m6A+ genes displayed a significant higher ratio of translation compared to m6A- genes or non-inherited m6A+ genes (new Fig S2C).*

3. The phenotype of YTHDC1 KD using Cas13d is not quite identical to the YTHDC1 KO in zygote in previously published paper2 (Chuan Chen et al., Protein & Cell, 2021) which have normal blastocyst rate but hatching problems. What's the differences? Did you test the protein level of YTHDC1 after KD assay?

*Response: We thank the reviewer for the constructive questions. In response to the reviewer’s suggestion, we further analyzed the previous study and noticed that they used Cas9 to knock out Ythdc1 while we used Cas13d. As previously shown in Fig S3B, compared to its transcription, Ythdc1 displayed a more dramatic translation pattern. It is highly possible that the maternally inherited part of Ythdc1 mRNA can play a role in preimplantation development, but these mRNAs cannot be depleted by Cas9. To test this, we also used Cas9 to knock out Ythdc1 and found that Ythdc1 KO did not affect blastocyst formation but significantly reduced hatching ratios (new Fig S3F and 3G).*

*In addition, we also performed Western blotting to measure the protein levels of Ythdc1 after KD. As illustrated in Fig S3H, an evident decrease of Ythdc1 protein level was observed at late 2-cell stages.*

4. METTL3 was recently found to ensure the timely decay the 2C-specific m6A transcripts according to Wu et al .1 Several studies about the m6A function in embryos and embryonic stem cells are recently published1-3. Citation in the manuscript need to be updated.

Reference:

1. Wu, Y. et al. N(6)-methyladenosine regulates maternal RNA maintenance in oocytes and timely RNA decay during mouse maternal-to-zygotic transition. Nat Cell Biol 24, 917-927, doi:10.1038/s41556-022-00915-x (2022).

2. Chen, C. et al. Nuclear m(6)A reader YTHDC1 regulates the scaffold function of LINE1 RNA in mouse ESCs and early embryos. Protein Cell 12, 455-474, doi:10.1007/s13238-021-00837-8 (2021).

3. Wei, J. et al. FTO mediates LINE1 m(6)A demethylation and chromatin regulation in mESCs and mouse development. Science 376, 968-973, doi:10.1126/science.abe9582 (2022).

*Response: Thanks for the kind reminder. We have cited these elegant papers and added discussion associated with these papers.*

Reviewer #2:

The manuscript "Reading and writing of mRNA m6A modification orchestrate mouse 2 maternal-to-zygotic transition" is a good effort to map changes in epitranscriptomic mark, m6A on maternal and zygotic RNA to understand the role of most prevalent RNA modification in zygotic genome activation. Particularly, authors have used the low input multi-omics strategies to reveal role of m6A during early phase of development. The low input multi-omics approaches can solve problem of the material required to study early development in mammals. Maternal-to-zygotic (MZT) transition involves enormous changes in the transcriptomic landscape, thus, it is plausible that the epitranscriptomic marks will change too and can play pivotal role in MZT. So far, no one has studied this systematically except one recent paper (Wu NCB 2022).

Here, the authors report the following conclusions:

1) Maternal m6A is inherited.

2) Newly transcribed mRNA in zygotes are m6A methylated.

3) Ythdf2 mediates m6A's function in mRNA decay and clearance.

4) Besides mediating mRNA degradation, m6A mediates mRNA stabilisation and promotes mRNA translation.

5) Ythdc1 has a role in stabilizing m6A methylated mRNAs. The conclusions of this manuscript are either purely based on correlation (4,5) or not notable (1-3). I will expand on my criticism of these conclusions.

*Response: We thank the reviewer for the helpful comments. We have performed additional bioinformatic and experimental analysis and included new results according the reviewer’s suggestions. We hope the reviewers and the editor will agree that our analyses and findings have been greatly improved and provided a better understanding of the m6A epitranscriptomic mark during MZT.*

Maternal m6A is inherited: There is poor evidence showing that m6A is demethylated after entering the cytoplasm since any potential demethylase is secluded to the nucleus. Therefore, if maternal mRNAs are inherited, it is not surprising that maternal m6A is also inherited.

*Response: We agree with the reviewer that it is not surprising that maternal m6A is inherited, but we still believe it is meaningful to investigate what these m6A-tagged genes are and how their fates would be after fertilization remain largely unknown. Our study has revealed that 435 maternally m6A+ genes lost their modification after fertilization which may due to m6A demethylation or mRNA degradation. However, we also found 609 genes are consistently inherited m6A mark.*

Newly transcribed mRNA in zygotes are m6A methylated: Since Mettl3 is present in zygotes, it is not surprising that newly transcribed mRNAs gain m6A. This is akin to any newly transcribed mRNA being spliced or gaining a mRNA cap.

*Response: We thank the reviewer for the meaningful comment. Even though Mettl3 is present in zygotes and we observed active transcription and translation of Mettl3 after fertilization (Fig S2A and S2B), it is important to reveal that whether or not it is ubiquitously modified on every newly transcribed mRNA. Here, we not only confirmed that newly transcribed mRNAs are m6A methylated, but also identified what they are and their associated functions, which deepened our understanding of the early selective m6A modification of mRNAs. This comment helped us to improve description and discussion of our manuscript.*

Ythdf2 mediates m6A's function in mRNA decay and clearance: This has already been demonstrated in mice (Ivanova 2017 Mol. Cell).

*Response: We thanks the reviewer for this great point. This excellent work was cited in our manuscript as well. In our present study, we included Ythdf2 to investigate due to the following three reasons.*

*1) Ythdf2 is an important reader for m6A metabolism as established by Ivanova et al. (Mol Cell, 2017).*

*2) We observed a significant upregulation of Ythdf2 at transcription, translation and protein levels after major ZGA which is unknown.*

*3) In the previous work (Mol Cell, 2017), the authors demonstrated that Ythdf2 is essential for preimplantation development using CKO mice, however they also showed that loss of Ythdf2 impaired oocyte maturation.*

*Thus, how does Ythdf2 expression at major ZGA affect preimplantation development is unknown. In short, our data discriminated the roles of Ythdf2 in oocyte maturation and early development. We have added the discussion in our revised manuscript.*

Besides mediating mRNA degradation, m6A mediates mRNA stabilisation and promotes mRNA translation: The evidence that the authors provide here are simply only correlative. The authors did not attempt any reverse genetics of m6A readers or Mettl3 to probe the effect of m6A on mRNA translation. Instead, they only show that for a subset of m6A mRNA, there is correlation between presence of m6A and active translation. Furthermore, the proteomics result are similar to other studies (Gao et al., Cell reports 2017) and here, they found no new hits.

*Response: We thank the reviewer for the kind questions. As also suggested by Reviewer 1, we have provided more bioinformatic analysis regarding this point. In addition, we knocked down Ythdc1 and found that loss of Ythdc1 reduced the mRNA levels of 226 m6A marked genes (New Fig 2N and 2O).*

*As for the proteomics data, we believe that there are at least two improvements that provided here.*

*1) Our proteomics data synchronized with our m6A-seq data, i.e., MII, late 1-cell (L1C) and late 2-cell (L2C). The previous study lacks MII stage and contains generally defined 1-cell and 2-cell stages.*

*2) Our proteomics data are more informative than the previous one. Totally 5328 proteins are reproducibly identified from the early three developmental stages, while the previous study reproducibly identified totally 3767 proteins from the whole preimplantation development (from zygote to blastocyst).*

3) Utilizing the proteomic data, we provided evidence to show that there are about 300 detected maternally inherited m6A+ mRNAs are maintained or increased during MZT (new Fig 2G). This finding cannot be made without the proteomic data from MII oocytes.

Ythdc1 has a role in stabilizing m6A methylated mRNAs: Past published results have only demonstrated that IGF2BP proteins mediate stabilisation of m6A mRNA. While the authors did knock down Ythdc1, they focused only on the subset of 44 down-regulated genes out of 376 genes that are both differentially expressed and possess m6A, in order to reach the conclusion that Ythdc1 functions in stabilizing these m6A mRNAs. At the very least, they will need some kind of crosslinking-IP experiment to show that Ythdc1 binds to these m6A mRNAs to stabilize them, which was not performed.

*Response: We thank the reviewer for the kind questions. First, we acknowledged that our previous data (old Fig 2P) are quite misleading, as the Reviewer 1 had the same concern. Previously, we mainly focused on the impact of Ythdc1 KD on a small group of mRNAs, i.e., de novo gained m6A. Among the down-regulated genes, there are 226 genes are marked with m6A (new Fig 2L and 2M) which are more than 44 genes.*

*According to the reviewer’s suggestion, we performed RNA immunoprecipitation (RIP)-qPCR assay based several selected candidates from the above-mentioned 226 genes. As shown in new Fig 2N and Fig S4D, m6A-marked mRNAs, including Ybx3, Rab3b, Commd3, Camk1d and Tcea18, are associated with Ythdc1. Additionally, qPCR further validated that knockdown of Ythdc1 reduced the mRNA levels of these genes (new Fig 2O and Fig S4E).*

*Collectively, we concluded that Ythdc1 is associated with m6A marked genes and involved in their stability regulation. Very recently, a study also demonstrated that YTHDC1 can modulate autophagy via regulating the stability of SQSTM1 mRNA in diabetic keratinocytes (Liang et al., Autophagy, 2022).*

With the following major concerns, I believe that in present form the manuscript is not suitable for Genome Biology.

Other comments: 1-The sample size to infer most results is low: 50 oocytes or embryos per replicates are not sufficient to make mechanistic conclusions.

*Response: We thank the reviewer for the question. As the reviewer mentioned above, our proteomics results are similar to a published study (Gao et al., Cell reports 2017). We have provided more comparative analysis to show that our proteomics data are as good as the previous one which used ~16,000 MII oocytes and ~32,000 early embryos (1C and 2C). As shown in Fig S2E, the majority of the identified proteins (2796/3767, ~74.2%) from large samples can be detected in our low-input proteomic data. Of note, there are another 2532 proteins are detected in our study. Therefore, with the development of ultra-sensitive proteomic technology in recent years, we believe our proteomics data are sufficient to make mechanistic conclusions.*

2-In addition, it will be great if authors can compare the results of LiRibo-seq with a recently published study where they profiled mRNA translation during mouse MZT (Zhang et al,. Science Adv. 2022) and low input m6A transcripts result with another recent study Wu et al,. 2022 (NCB).

*Response: We thank the reviewer for the questions. It seems that the Reviewer missed the information that the LiRibo-seq data used in our analysis are indeed from Zhang et al, (Sci Adv, 2022).*

*As the reviewer and Reviewer 1 suggested, we have compared our results with the most recently published m6A data (Wu et al., NCB, 2022). Even though the two studies used different mA6-seq methods (SLIM-seq vs ULI-MeRIP-seq), sample numbers (100~250 oocytes or embryos per sample vs 540~750 oocytes or embryos per sample), mice background (B6D2F1 vs C57BL/6) and ways of fertilization (in vivo vs in vitro), we both identified a large number of the same genes with m6A marker (new Fig S1B and S1C). The PCA analysis further verified a similar pattern of the m6A dynamics during MTZ (new Fig S1D), even though a batch effect is observed (Dim 2).*

*In short, the two studies generally confirmed each other independently, but the transcriptomic data obtained from different experimental conditions and methods showed evident batch effects that rendered their in-depth comparison.*

3-The legends should be consistent and the presentation of results should be coherent, at least in the same figure. Inconsistent legends are bothersome for the readers and difficult to compare. For example in Figure 1B for m6A-Seq, the sequence of the results are arranged differently than RNA Seq.

*Response: We apologize for the mistakes and appreciate the constructive suggestions. We have rearranged our data and figure legends, and made sure they are coherent in the revised manuscript.*

4-The quality of the figures should be improved and presentation should be clear. Moreover, the resolution of figures, particularly Figure 2 is quite low.

*Response: We apologize for the inconvenience. The resolution of our figures was compressed by the online system which we could avoid. We have fixed these issues in the revised version.*

**Second round of review**

**Reviewer 1**

The authors have addressed all the questions raised by this reviewer.

**Reviewer 3**

In this manuscript of Zhu et al. try to make m6A map changes on maternal and zygotic RNA to understand the role of RNA m6A modification in zygotic genome activation. This study is of great interest and novelty, whereas in this revised manuscript they might not provide enough evidence to address concerns from Reviewer #2.

The authors claim that 609 maternal m6A+ genes are consistently inherited. How many of the m6A- genes are inherited? Are m6A+ genes more likely, less likely or similarly inherited as m6A- genes?

The authors need to provide evidence or control to prove that their SLIM-seqs were well done. The "inherited" genes show generally lower m6A level and stronger variation between repeats than "maternal loss" or "De novo gain" genes. It is possible that these genes also underwent m6A changes which is not detected due to the sensitivity of the m6A detecting method. It will be helpful to add synthesized oligos as spike in to validate the sensitivity of the m6A detecting assay used in this study.

SLIM-seq data analysis methods described in the methods seems confusing. According to the authors’ description “The read counts for each gene were assessed by R package tximport (v1.20.0)”. Total reads were normalized? I think this is not a good choice. This analysis method is also different from the Ref #20 the authors cited.

How many newly transcribed mRNAs do not gain m6A in the zygotes?

As the authors observed both up-regulated genes and down-regulated genes upon YTHDC1, and most of these genes are not detected with m6A, it is interesting to ask how many of these genes in are the direct target of YTHDC1 (bound by YTHDC1)? Is it dependent on the m6A reading ability of YTHDC1? Does YTHDC1 regulates the transcription process, degradation process or both of the target genes? There seems no evidence to prove that YTHDC1 plays a role to stabilize RNA.

**Reviewer 4**

Zhu et al. have substantially revised their manuscript. Specifically, they have now provided additional bioinformatical analyses regarding the m6A modification dynamics and its correlation to mRNA translation/stability during MZT. They have also provided additional evidence to validate the role of Ythdc1 in mRNA stability, as well as a better discussion about their findings comparing to the recent study about m6A modification during MZT. Overall, the authors have done a good job in responding to most of the reviewers’ comments.

However, with the new information provided in the revised manuscript, some additional concerns are raised:

1) In Figure 1C, the authors showed that the numbers of m6A-tagged mRNAs significantly decreased from MII to L1C (2153 to 1213). However, Wu et al., showed that m6A+ transcripts and peaks increased after fertilization (Wu et al., Nat Cell Biol, 2022). In addition, the ULI-MeRIP-seq can detect much more m6A+ transcripts than SLIM-seq. Is there any good explanation for these inconsistency?

2) In Figure 1G, to get an overview of the correlation between m6A modification and mRNA expression, it’s better to show the heatmap of all of the 982 MII lost and 1243 L2C de novo gained genes.

3) The current results are not enough to validate the function of Ythdc1 function in mRNA stability. The evidence they showed here is based on the correlation between RNA-seq and Ythdc1 binding. It’s better to have a mRNA stability assay with Actinomycin D treatment.

4) In addition, The authors claimed that Ythdc1 prefers to maintain RNA stability for m6A-marked genes based on the results showing that about 10% of down-regulated genes are marked with m6A after Ythdc1 knock-down. However, among the up-regulated genes, there are also about 10% of genes marked with m6A, the authors should provide an explanation.

5) Furthermore, to explain the inconsistent phenotype in Ythdc1 knockdown and knockout, a western blot is suggested to show the protein changes after Ythdc1 knockdown and knockout.

Minor comments:

1) The statement in Discussion “we revealed and confirmed that a group of maternal inherited m6A maintains mRNA stability and promotes translation” is only based on correlation, especially for “promotes translation”. The authors may need to reconsider this statement.

2) In page 3 Line 51, an extra “a” is in “A key question for embryonic development is a how MZT process is regulated”.

**Authors Response**

**Point-by-point responses to the reviewers’ comments:**

Reviewer #1: The authors have addressed all the questions raised by this reviewer.

*Response: We appreciate the Reviewer’s helpful comments that have improved our manuscript.*

Reviewer #3:

In this manuscript of Zhu et al. try to make m6A map changes on maternal and zygotic RNA to understand the role of RNA m6A modification in zygotic genome activation. This study is of great interest and novelty, whereas in this revised manuscript they might not provide enough evidence to address concerns from Reviewer #2.

The authors claim that 609 maternal m6A+ genes are consistently inherited. How many of the m6A- genes are inherited? Are m6A+ genes more likely, less likely or similarly inherited as m6A- genes?

*Response: Thanks for the comments. In response to the reviewer’s question, we have calculated the number of inherited mRNA genes among untagged m6A genes. Comparison of the inherited genes between m6A+ and m6A- groups showed that a higher ratio of inherited genes in m6A tagged genes, indicating that m6A+ genes are more likely to be inherited than m6A- genes (new Figure S2H).*

The authors need to provide evidence or control to prove that their SLIM-seqs were well done. The "inherited" genes show generally lower m6A level and stronger variation between repeats than "maternal loss" or "De novo gain" genes. It is possible that these genes also underwent m6A changes which is not detected due to the sensitivity of the m6A detecting method. It will be helpful to add synthesized oligos as spike in to validate the sensitivity of the m6A detecting assay used in this study.

*Response: We apologized for the misleading information presented in Figure 1G of our original manuscript. The expression level was z-score scaled for each gene, and colors represent the relative expression for each gene among different samples. Therefore, the colors in the heatmap are not comparable for different genes. To address the reviewer’s concern, we further compared the expression levels (log2 normalized TPM without z-score scale) for "maternal loss", "De novo gain" and “Inherited” mRNA genes at different developmental stages. The results showed that inherited genes showed comparable m6A levels to the “maternal loss” or “De novo gain” genes (new Figure S2G).*

*As suggested by the Reviewer, we add synthesized mRNAs as spike-in to validate the sensitivity of the m6A detecting method. We used 50 ng of total RNA from MII oocytes, and added m6A+ GFP mRNAs and m6A- mCherry mRNAs at 1:1000 dilution. The MeRIP-qPCR results exhibited relatively high signal-to-noise ratios of spike-in mRNAs GFP/mCherry (new Figure S1A), indicating the high sensitivity and specificity of the method.*

SLIM-seq data analysis methods described in the methods seems confusing. According to the authors’ description “The read counts for each gene were assessed by R package tximport (v1.20.0)”. Total reads were normalized? I think this is not a good choice. This analysis method is also different from the Ref #20 the authors cited.

*Response: We apologize for the confusing description in the Methods of our original manuscript. Actually, we applied the same methods mentioned in the reference paper (Yin R, Chang J, Li Y, et al. Cell Stem Cell. 2022) for analyzing our SLIM-seq data. Specifically, R package tximport (Soneson C, Love MI and Robinson MD. F1000Research 2016) was used to estimate the read counts at gene level from the alignment files obtained by kallisto. Then, we normalized the read counts for each gene by total reads in each sample and gene length to eliminate the effect of different library sizes, and transformed counts matrix to TPM matrix. The TPM values were used for the following analysis. We have added detailed descriptions in the Methods of revised manuscript.*

How many newly transcribed mRNAs do not gain m6A in the zygotes?

*Response: Thanks for the question. Among 3031 newly transcribed mRNAs (i.e., major ZGA genes), 428 mRNAs were tagged with m6A, and 2603 mRNAs do not gain m6A (new Figure S1F).*

As the authors observed both up-regulated genes and down-regulated genes upon YTHDC1, and most of these genes are not detected with m6A, it is interesting to ask how many of these genes in are the direct target of YTHDC1 (bound by YTHDC1)? Is it dependent on the m6A reading ability of YTHDC1? Does YTHDC1 regulates the transcription process, degradation process or both of the target genes? There seems no evidence to prove that YTHDC1 plays a role to stabilize RNA.

*Response: We thank the reviewer for these open questions, and we are glad to know that the reviewer is interesting in our work. We preliminarily conclude that Ythdc1 plays a role to stabilize a small group of maternal inherited mRNAs with m6A mark based on the following evidence.*

*1) Knockdown of Ythdc1 leads to down-regulation of a group of genes with m6A mark as evidenced by RNA-seq and SLIM-seq data.*

*2) Using RIP-qPCR, we validated the binding of Ythdc1 of several mRNAs with m6A mark.*

*3) As suggested by Reviewer 4, we further performed a mRNA stability assay with Actinomycin D treatment and consolidated that Ythdc1 is involved in the stability regulation of maternal transcripts of these genes (new Fig. S3D-E). The data support a role of Ythdc1 in stabilizing mRNA, and excluded the transcription process.*

*4) A recent study also demonstrated that YTHDC1 can modulate autophagy via regulating the stability of SQSTM1 mRNA in diabetic keratinocytes (Liang et al., Autophagy, 2022).*

*We believe that more future efforts are required to fully uncover the roles of Ythdc1 played in regulation of RNA fate. The related discussion is added into our revised manuscript.*

Reviewer #4:

Zhu et al. have substantially revised their manuscript. Specifically, they have now provided additional bioinformatical analyses regarding the m6A modification dynamics and its correlation to mRNA translation/stability during MZT. They have also provided additional evidence to validate the role of Ythdc1 in mRNA stability, as well as a better discussion about their findings comparing to the recent study about m6A modification during MZT. Overall, the authors have done a good job in responding to most of the reviewers’ comments.

However, with the new information provided in the revised manuscript, some additional concerns are raised:

1) In Figure 1C, the authors showed that the numbers of m6A-tagged mRNAs significantly decreased from MII to L1C (2153 to 1213). However, Wu et al., showed that m6A+ transcripts and peaks increased after fertilization (Wu et al., Nat Cell Biol, 2022). In addition, the ULI-MeRIP-seq can detect much more m6A+ transcripts than SLIM-seq. Is there any good explanation for these inconsistency?

*Response: We thank for the reviewer’s question. We did observe that there was a batch effect between the two mA6-seq methods (SLIM-seq vs ULI-MeRIP-seq), and the ULI-MeRIP-seq detected more genes than our currently conducted SLIM-seq method. We speculate that the inconsistency between the two methods might be caused by the following reasons.*

*First, Wu et al. collected mRNAs from 540~750 oocytes or embryos per sample for sequencing, while we collected 100~250 oocytes or embryos for each sample. The number of genes detected in each sample was positively correlated with the number of oocytes or embryos included.*

*In addition, the mice background (B6D2F1 in our study vs C57BL/6 in the literature), ways of fertilization (in vivo vs in vitro) and sequencing depth are different between the two studies.*

2) In Figure 1G, to get an overview of the correlation between m6A modification and mRNA expression, it’s better to show the heatmap of all of the 982 MII lost and 1243 L2C de novo gained genes.

*Response: We appreciate for the reviewer’s suggestion, and have showed the heatmap of all of the 982 MII lost and 1243 L2C de novo gained genes in updated Figure 1G of revised manuscript.*

3) The current results are not enough to validate the function of Ythdc1 function in mRNA stability. The evidence they showed here is based on the correlation between RNA-seq and Ythdc1 binding. It’s better to have a mRNA stability assay with Actinomycin D treatment.

*Response: Thanks for the constructive suggestion. As shown in new Fig S3C-D, knockdown of Ythdc1 started to work at 4 hours post microinjection and the Actinomycin D treatment is effective as Ythdc1 should be upregulated at L1C stage (PN5) (Fig S4A). More importantly, the mRNA stability of these tested genes is evidently reduced after the knockdown of Ythdc1 (new Fig S3E).*

4) In addition, the authors claimed that Ythdc1 prefers to maintain RNA stability for m6A-marked genes based on the results showing that about 10% of down-regulated genes are marked with m6A after Ythdc1 knock-down. However, among the up-regulated genes, there are also about 10% of genes marked with m6A, the authors should provide an explanation.

*Response: Thanks for the question. We think this is a consequence after Ythdc1 KD-induced dysregulation. Although these upregulated genes contain m6A mark, it does not mean that the m6A modification levels on these mRNAs are increased or that Ythdc1 participates in the stability regulation of these genes since there are several m6A reader proteins. Similar to the recent report (Wu et al., Nat Cell Biol, 2022), conditional knock-out of Kiaa1429, a key component of m6A writer complex, could result in up-regulation of some m6A-tagged transcripts. We have added some discussion in our revised manuscript.*

5) Furthermore, to explain the inconsistent phenotype in Ythdc1 knockdown and knockout, a western blot is suggested to show the protein changes after Ythdc1 knockdown and knockout.

*Response: Thanks for the suggestion. We performed the western blot, and our results confirmed our previous speculation that Cas13d-mediated knockdown is more efficiently than Cas9-mediated knockout to lower the protein levels of Ythdc1.*

Minor comments:

1) The statement in Discussion “we revealed and confirmed that a group of maternal inherited m6A maintains mRNA stability and promotes translation” is only based on correlation, especially for “promotes translation”. The authors may need to reconsider this statement.

*Response: Thanks, we have rephased this statement.*

2) In page 3 Line 51, an extra “a” is in “A key question for embryonic development is a how MZT process is regulated”.

*Response: Thanks, we have corrected it.*

**Third round of review**

**Reviewer 3**

I appreciate the authors provided much more data to address my concerns and believe this maunscript is a strong candidate for journal of Genome Biology.

**Reviewer 4**

The authors have addressed all the concerns raised by this reviewer.

**Authors Response**

**Point-by-point responses to the reviewers’ comments:**

Reviewer #3

I appreciate the authors provided much more data to address my concerns and believe this maunscript is a strong candidate for journal of Genome Biology.

Reviewer #4

The authors have addressed all the concerns raised by this reviewer.

*Response: We are pleased to hear that the reviewers are satisfied with our revised manuscript and we appreciate their valuable input.*
